# Design of Eutectic Solvents with Specified Extraction Properties Based on Intermolecular Interaction Energy

**DOI:** 10.3390/molecules29215022

**Published:** 2024-10-24

**Authors:** Arina V. Kozhevnikova, Ekaterina S. Uvarova, Varvara E. Maltseva, Ivan V. Ananyev, Nikita A. Milevskii, Igor S. Fedulov, Yulia A. Zakhodyaeva, Andrey A. Voshkin

**Affiliations:** 1 Kurnakov Institute of General and Inorganic Chemistry, Russian Academy of Sciences, 119991 Moscow, Russia; ak@igic.ras.ru (A.V.K.); uvarovacatya@yandex.ru (E.S.U.); varyamalceva@mail.ru (V.E.M.); i.ananyev@gmail.com (I.V.A.); mna@igic.ras.ru (N.A.M.); yz@igic.ras.ru (Y.A.Z.); 2 Institute for African Studies, Russian Academy of Sciences, 123001 Moscow, Russia; if345@ya.ru

**Keywords:** hydrophobic eutectic solvents, solvent extraction, aliphatic alcohols, selectivity, metal extraction

## Abstract

A new approach to managing the extraction properties of eutectic solvents based on aliphatic alcohols is proposed. Aliphatic alcohols, when functioning as hydrogen bond donors within a eutectic solvent, significantly enhance the solvent’s efficiency in extracting metal ions. Conversely, when the alcohol acts as a hydrogen bond acceptor, its extraction properties diminish. Molecular modelling reveals that the extraction efficiency of these alcohols is directly proportional to the intermolecular interaction energy between the components of the eutectic solvent.

## 1. Introduction

Deep eutectic solvents (DESs) represent a relatively new generation of extractants, composed of a hydrogen bond donor (HBD) and a hydrogen bond acceptor (HBA). Their applications span various fields, including medicine [1,2] and analytical chemistry [3,4,5]. DESs are used as solvents for the synthesis of organic [6,7] and inorganic compounds [8]. They also show promise as extractants for the extraction and separation of both organic [9,10,11] and inorganic compounds [12,13,14,15,16,17,18,19]. Depending on their miscibility with water, DESs are classified as either hydrophilic [20] or hydrophobic (hydrophobic deep eutectic solvents (HDESs)) [21]. Initially, defining this class of extractants posed a challenge, prompting researchers to explore various combinations of components and study their physical properties.

The first generation of eutectic solvents was based on mixtures of quaternary ammonium salts with hydrogen bond donors. However, the classification of DESs has since expanded to four distinct classes [22]. A major breakthrough in DES research was the discovery of a fifth type, Type V [23], prepared exclusively from non-ionic compounds. The diversity of these non-ionic components has opened up new possibilities for synthesising DESs tailored to specific tasks. This paper focuses on HDESs, presenting findings related to the extraction of metals from aqueous solutions using these solvents.

Despite their novelty, a substantial body of research on HDESs, which has featured their composition and characteristics, has already been established [24,25]. Recent studies have increasingly focused on understanding the nature of interactions between the hydrogen bond donor and acceptor within the mixture, which largely influences properties such as water solubility, density, viscosity and the eutectic position on the solid–liquid phase diagram [26]. For instance, the molecular modelling of various HDESs has been conducted to predict key physical properties relevant to the extraction process, including density, phase behaviour, water solubility and the efficiency of organic acid extraction [27]. Furthermore, there is growing interest in studying the intermolecular forces within HDESs by comparing calculated and experimental phase diagrams. Abranches et al. [28] investigated the potential for forming HDESs using fatty alcohols and acids. Their findings indicated that thymol (Thy) facilitates DES formation by inducing negative deviations from the ideal in the phase diagram, while choline chloride was unable to form DESs with either fatty acids or alcohols.

There have, however, been relatively few studies that have both examined the physical properties of HDESs and explored how the nature of their components and the interactions between them influence extraction efficiency. A limiting factor in the use of HDESs for extraction is the decreased extractive ability of the extractant, which is primarily due to the hydrogen bonds and van der Waals interactions formed between the donor and acceptor. For example, the extraction of Pt(IV) and Pd(II) ions using HDESs based on trioctylphosphine oxide (TOPO) has been studied [29]; the study revealed that metal extraction is complex, involving an antagonism between the mechanism of interphase distribution and the intermolecular interactions of the HDES components. It was also established that substituting thymol for decanoic acid as the HBD reduces metal extraction efficiency, as thymol forms a more extensive hydrogen bond network with TOPO than decanoic acid, hindering the formation of an extractable metal complex.

In a different study [30], the extraction ability of three TOPO-based HDESs as the HBAs and with phenol, para-tert-butylphenol, and thymol as the HBDs was investigated. It was shown that the second component, which lacked inherent extraction properties, significantly enhanced the extractability of Sm(III) ions in the order thymol/TOPO < para-tert-butylphenol/TOPO < phenol/TOPO. This increase in extractability was attributed to a decrease in the interaction energy between the donor and acceptor in this order. Thus, varying the nature of the second component enables control of HDES extraction properties.

**Figure 1:**
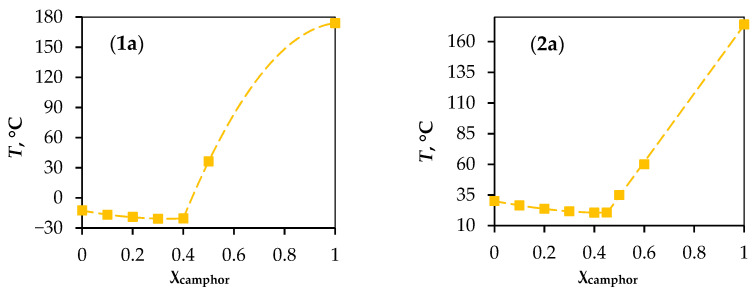

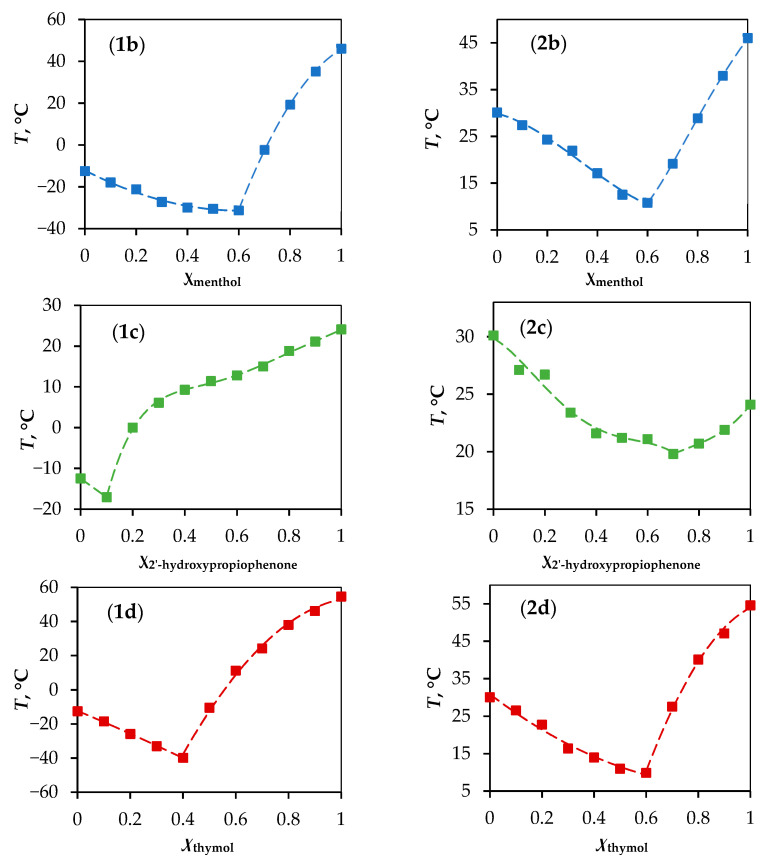
Phase diagrams of HESs based on 1-octanol (**1**) and 1-dodecanol (**2**) with (**a**) camphor, (**b**) menthol, (**c**) 2′-hydroxypropiophenone, (**d**) thymol.

The purpose of this work was to establish the dependence of the extraction ability of hydrophobic eutectic solvents (HESs) concerning metals on their composition. We omit the characteristic “deep” since not all the studied eutectic solvents correspond to it. In this work, the name HES was established in accordance with the classification introduced in the work of Abranches et al. [31]. To this end, the extraction ability of ten HESs based on aliphatic alcohols with respect to Fe(III) ions was studied. Iron was chosen as a model due to its prevalence in complex separable mixtures. The aliphatic alcohols 1-octanol (Oct) and 1-dodecanol (Dod) were used as the primary components, while camphor (Cam), 2′-hydroxypropiophenone (Hpph), L-menthol (Men), 1-octanoic acid (OctA), and thymol were selected as second components. It is important to note that these components exhibited little to no extraction ability for Fe(III) ions under the experimental conditions. The alcohol-to-second component ratio in the HESs in the extraction experiments was 7:3, as all HESs studied at this ratio are liquid (Figure 1). This ratio was chosen to compare the extraction properties of eutectic solvents at the same alcohol concentration, which is important.

## 2. Results and Discussion

The formation of HESs was confirmed using Fourier infrared spectroscopy and ^1^H NMR spectroscopy. For this purpose, the spectra of the initial compounds and HESs based on them were recorded. The shift in the peak of the stretching vibration of the -OH group of alcohol (1-octanol and 1-dodecanol) and the shift in the peaks of vibrations of the functional groups of the second component in the HESs are shown in the obtained IR spectra (Figures S1–S10). The characteristic shifts are indicated in the figures. The obtained results indicate the formation of hydrogen bonds between the components in the HESs [32,33]. Phase diagrams of the mixtures were also constructed (Figure 1), except for the HESs based on 1-octanoic acid, for which phase diagrams were provided in a previous study [33]. The data confirm that the proposed solvents exhibit a eutectic phase diagram. It should also be mentioned that these HESs have a fairly wide range of liquid state, which allows varying the required amount of aliphatic alcohol.

After confirmation of the formation of HESs, their extraction ability for iron ions was studied. It is known that aliphatic alcohols form self-associates, which diminishes their metal extraction efficiency. Dilution with aromatic hydrocarbon solvents helps to break these self-associations [34]. In the composition of HESs, aliphatic alcohols can function as both HBDs and HBAs, which depends on whether the oxygen or hydrogen from the OH group of the alcohol participates in the formation of the hydrogen bond, which in turn depends on the nature of the second component participating in the interaction with the alcohol. In the case where a hydrogen atom is involved in the formation of the hydrogen bond, the alcohol acts as a hydrogen bond donor. Similarly, if the oxygen of the OH group participates in the formation of the hydrogen bond, the alcohol is a hydrogen bond acceptor. Thus, depending on whether the alcohol is an HBA or an HBD, its extraction properties presumably change. This study analysed the changes in iron extraction efficiency with 1-octanol/1-dodecanol HESs, depending on the nature of the second component, by comparing the extractability of iron with the corresponding alcohols dissolved in a nonpolar solvent (toluene).

The extraction of iron(III) from hydrochloric acid solutions was studied based on the composition of the aqueous phase and the HESs. The mechanism of iron extraction by aliphatic alcohols is described by the following equations [35]:

(1) $$\mathrm{ROH}+\mathrm{HCl}⇌{\left(\mathrm{ROH}\cdot H\right)}^{+}{\mathrm{Cl}}^{-},$$

where ROH—C_8_H_17_OH/C_12_H_25_OH.

(2) $${\left(\mathrm{ROH}\cdot H\right)}^{+}{\mathrm{Cl}}^{-}+H[{\mathrm{FeCl}}_{4}]⇌{\left({\mathrm{ROH}}_{2}\right)}^{+}{\left[{\mathrm{FeCl}}_{4}\right]}^{-}+\mathrm{HCl}$$

**Figure 2:**
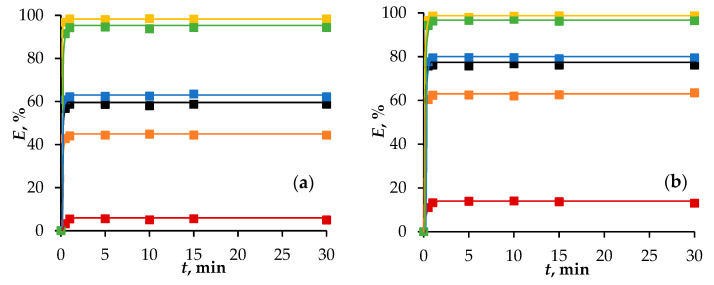
The extraction of Fe(III) ions depends on the time of phase contact with HESs based on (**a**) 1-octanol and (**b**) 1-dodecanol. Aqueous phase: [HCl] = 5 mol/L for 1-octanol-based HESs and [HCl] = 6 mol/L for 1-dodecanol-based HESs. Second component in HESs: 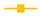
—camphor, 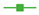
—2′-hydroxypropiophenone, 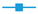
—menthol, 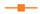
—1-octanoic acid, 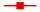
—thymol. 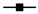
—1-octanol/1-dodecanol in toluene.

An important parameter in the extraction process is the time to reach thermodynamic equilibrium. This study of this parameter was carried out in the range of 0.5 to 30 min (Figure 2). The initial HCl concentrations of 5 and 6 mol/L were used for clarity and convenience of comparison. 1-Octanol-based eutectic solvents exhibit better extraction properties than 1-dodecanol-based eutectic solvents at the same HCl concentration. If we take the same HCl concentration, for example, 5 mol/L, then Dod/Thy does not extract Fe(III) ions, and the kinetic dependencies of the degree of extraction of Fe(III) ions is not clear. It was determined that 1 min was sufficient to reach thermodynamic equilibrium during the extraction of iron by the HESs under study. Therefore, a contact time of 5 min was used in all experiments.

**Figure 3:**
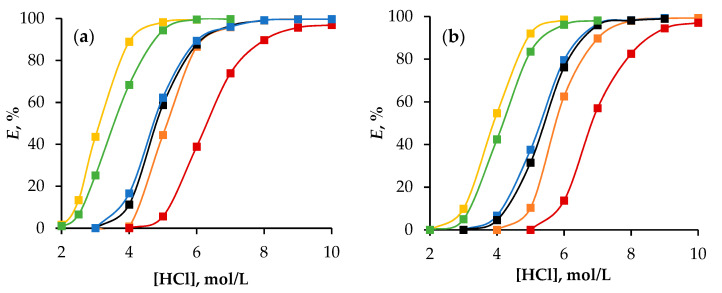
The extraction of Fe(III) ions depends on the concentration of HCl in a system with HESs based on (**a**) 1-octanol and (**b**) 1-dodecanol. The second component in HESs is 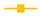
—camphor, 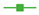
—2′-hydroxypropiophenone, 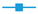
—menthol, 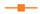
—1-octanoic acid, 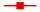
—thymol, 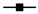
—1-octanol/1-dodecanol in toluene.

The effect of HCl concentration in the aqueous phase on the extraction efficiency of Fe(III) ions was studied (Figure 3). After extraction, the electronic spectra of the organic phase were recorded to confirm the reaction mechanism described by Equation (2) (Figures S11 and S12).

The spectra indicate that iron ions in the HES phase exist as anionic complexes, specifically [FeCl_4_]^−^. The increased degree of Fe(III) extraction with increasing HCl concentration can be attributed to the protonation of alcohol (ROH···H)^+^, which favours the formation of the [FeCl_4_]^−^ complex, thereby facilitating the reaction outlined in Equation (2). The results also indicate that 1-octanol is a more efficient extractant than 1-dodecanol. This difference in extraction efficiency is likely due to the decrease in the number of active functional groups per unit volume of the extractant that occurs as the length of the alkyl chain increases.

**Figure 4:**
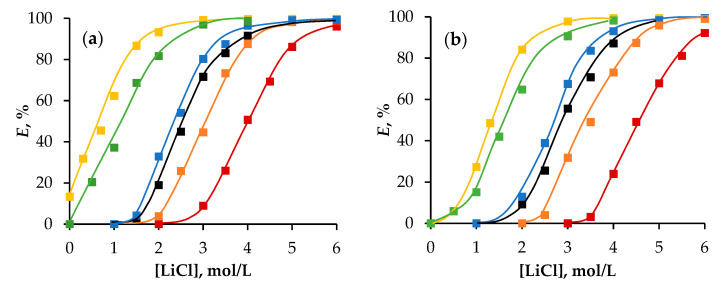
The extraction of Fe(III) ions depends on the concentration of LiCl in a system with HESs based on (**a**) 1-octanol and (**b**) 1-dodecanol. Aqueous phase: [HCl] = 2.5 mol/L. Second component in HESs: 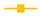
—camphor, 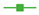
—2′-hydroxypropiophenone, 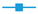
—menthol, 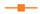
—1-octanoic acid, 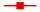
—thymol. 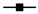
—1-octanol/1-dodecanol in toluene.

To further confirm the process described by mechanism (2), the effect of LiCl concentration in the aqueous phase was studied while maintaining a constant acidity of 2.5 mol/L HCl (Figure 4). The observed trends in iron extractability with increasing LiCl concentration closely mirrored those seen with varying HCl concentrations. As LiCl concentration increased, the degree of Fe(III) ion extraction also increased. The electronic spectra of the organic phase were recorded (Figures S13 and S14), confirming that, similar to previous findings, the iron ions in the HES phase exist as anionic complexes [FeCl_4_]^−^.

As shown in Figure 3 and Figure 4, the extraction efficiencies of all HESs varied, despite there being identical concentrations of 1-octanol and 1-dodecanol in their compositions (Table 1).

The efficiency of extraction improved in the order Oct/Men (Dod/Men) < Oct/Hpph (Dod/Hpph) < Oct/Cam (Dod/Cam) compared with aliphatic alcohol in toluene and decreased in the series Oct/OctA (Dod/OctA) > Oct/Thy (Dod/Thy). This indicates that the addition of camphor, 2′-hydroxypropiophenone, and *L*-menthol positively affects the extraction ability of the alcohol, while octanoic acid and thymol have a negative impact.

**Figure 5:**
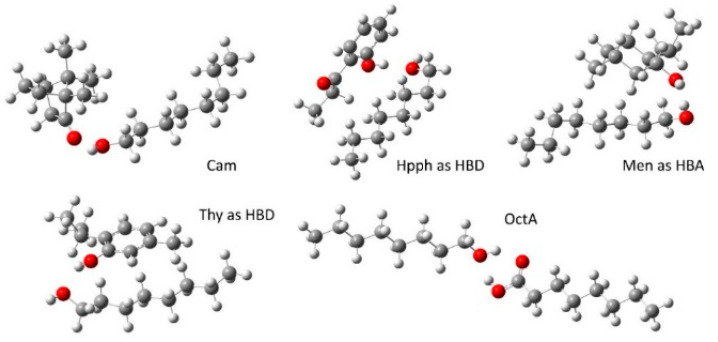
Relaxed structures of the most stable of octanol’s associates.

To better understand the influence of the second component on the extraction ability of the HESs, molecular modelling of the alcohol associates and the second components was conducted. Specifically, Born–Oppenheimer molecular dynamics modelling for two contrasting pairs—Oct/Cam and Oct/Thy (see Supplementary Information and Figures S15 and S16)—confirmed that the most stable supramolecular formations in liquid mixtures correspond to associates with a 1:1 component ratio and hydrogen bonds. Also, according to the combined DFT+D and DLPNO-CCSD calculations (Figure 5 and Tables S2–S16), in the most stable structures of Oct associates, the alcohol formally plays the HBD role in the complexes with Cam and Men and serves as an HBA in the context of Thy and Hpph. The dual role of octanoic acid should be mentioned here, as it forms two hydrogen bonds with the octanol molecule. There are, however, supramolecular conformers of the octanol’s complexes with Hpph, Men, and Thy in different roles (Figure S17 and Table S17).

**Figure 6:**
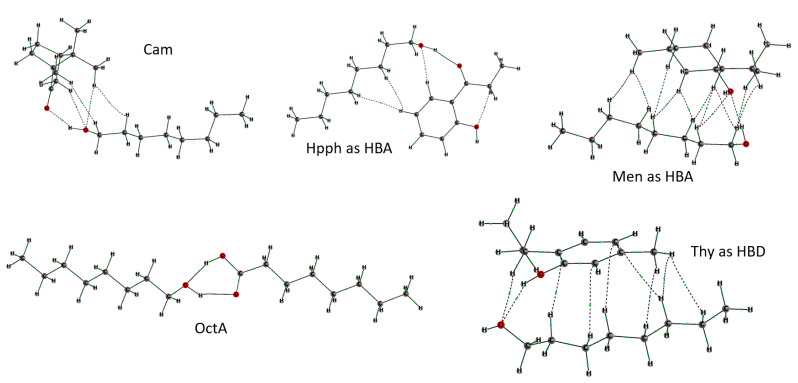
Atomic bonding graphs for selected associates of 1-octanol. Small green spheres denote (3,-1) critical points of electron density.

These structures are only slightly less stable than those in the global minima: the largest variation in Gibbs energy was observed for the associate of 2′-hydroxypropiophenone (1.5 kcal/mol), which can thus adopt the HBA role by itself. It should be noted that an analysis of bonding intermolecular interactions within the «Atoms in Molecules» framework [36] demonstrated the presence of other non-covalent interactions (such as CH···π and CH···O interactions), in addition to conventional hydrogen bonds, in nearly all associates (Figure 6).

**Figure 7:**
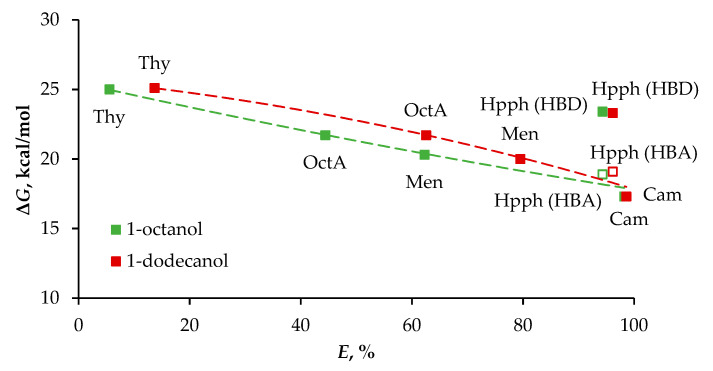
Correlation of the complexation energy with the degree of Fe(III) ion extraction. Aqueous phase for extraction: [HCl] = 5 mol/L for 1-octanol and [HCl] = 6 mol/L for 1-dodecanol.

With the sole exception of Hpph, the complexation energy for the most stable associates correlated well with the degree of Fe(III) ion extraction (Table 2 and Figure 7). Furthermore, considering the Hpph component as an HBA made it possible to obtain an excellent description of the extraction experiments with the same trend for dodecanol and octanol associates (see trend line in Figure 7). The general tendency of increasing extraction degree with decreases in the strength of intermolecular interaction supports mechanism (2), which implies the alcohol’s ability to be fully protonated. Estimates of bonding contributions to complexation energy suggest that the net energy of CH···π and CH···O interactions is comparable with that of hydrogen bonds (Table 2). Similarly, as in the case in Figure 2, at a concentration of HCl equal to 6 mol/L in the extraction with 1-octanol, the dependence of the degree of extraction of iron(III) ions on the energy of the complex (Figure 7) is uninformative, since both Oct/Cam and Oct/Hpph have the same degree of extraction of Fe(III) ions, which did not allow us to compare the contribution of Cam and Hpph to the extraction properties of the eutectic solvents.

In other words, the common paradigm for describing HESs’ extractive ability solely in terms of H-bonding features may fail in cases with hindered hydrocarbon substituents. This is in line with the almost-constant Bader’s atomic charge of the alcohol’s oxygen atom, which should have manifested the nucleophilic properties of an alcohol but decreased them only slightly, from −1.12e in the complexes of Thy to −1.17e in the Cam associates.

**Figure 8:**
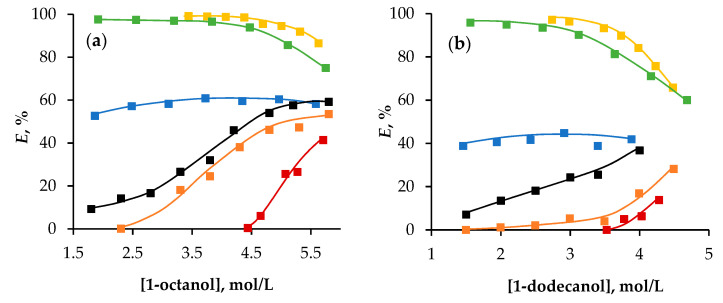
Dependence of the degree of extraction of Fe(III) ions on the concentration of 1-octanol/1-dodecanol in HESs based on (**a**) 1-octanol and (**b**) 1-dodecanol. Aqueous phase: [HCl] = 2.5 mol/L, [LiCl] = 2.5 mol/L. The second component in HESs is 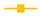
—camphor, 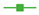
—2′-hydroxypropiophenone, 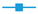
—menthol, 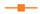
—octanoic acid, 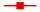
—thymol, 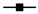
—1-octanol/1-dodecanol in toluene.

To further validate the proposed theory, the effect of alcohol concentration in HESs on the extraction behaviour of Fe(III) ions was studied within the range where the extractant remains in a liquid state (Figure 8). The data showed that for HESs containing OctA and Thy, the efficiency of the iron extraction increased with increasing alcohol concentration, similar to the behaviour of alcohol in toluene. This increase can be attributed to the higher alcohol-to-Fe(III) ratio, which shifts the equilibrium towards the formation of the extracted compound.

An atypical extraction pattern was, however, observed for the HESs containing Cam, Hpph, and Men. Specifically, the efficiency of iron extraction decreased when the alcohol concentration exceeded approximately 3.5 mol/L. This occurred because as the concentrations of Cam, Hpph, and Men decreased, the formation of Oct/Men (Dod/Men), Oct/Hpph (Dod/Hpph), and Oct/Cam (Dod/Cam) associates diminished, while the formation of Oct/Oct (Dod/Dod) associates increased. This shift led to a change in the hydrogen-bonding network, where the hydrogen bonds between the alcohol’s -OH group proton and oxygen were replaced by hydrogen bonds between two alcohol molecules. These alcohol–alcohol hydrogen bonds hindered the protonation of alcohol during extraction, thereby reducing the formation of the iron complex as described in Equation (2).

## 3. Materials and Methods

### 3.1. Reagents

Information on the reagents used in the experiments is presented in Table 3. All chemicals were used in the form in which they were received from the supplier, without additional purification.

### 3.2. Preparation of HESs

All HES mixtures were prepared by mixing an aliphatic alcohol (1-octanol/1-dodecanol) with a second component (camphor, 2′-hydroxypropiophenone, menthol, 1-octanoic acid, thymol) in a molar ratio of 7:3, unless otherwise noted. The mixture of HBA and HBD was stirred in a temperature-controlled shaker (Enviro-Genie SI-1202, Scientific Industries, Inc., Bohemia, NY, USA) (accuracy of ±0.2 °C), at 60.0 °C for 30 min, until a homogeneous transparent liquid was formed. After the preparation, the formed liquid was cooled slowly to room temperature and stored in air until use.

### 3.3. Characterisation of HESs

FT-IR spectra in the range of 4000–600 cm^−1^ were recorded on an IRTracer-100 spectrometer (Shimadzu, Tokyo, Japan) with a diamond crystal ATR accessory. The densities of the HESs were determined in the temperature range of 15–60 °C using an Anton Paar DMA 1001 (Anton Paar, Graz, Austria) with a precision of ±0.0001 g·cm^−3^. Based on the obtained densities, the concentrations of 1-octanol and 1-dodecanol in HESs were calculated, which are listed in Table 1.

The HES phase diagram was constructed using the DSC method on a DSK-500 instrument (Samara, Russia). For this, at least nine samples were prepared, covering the range of mole fractions from 0.1 to 0.9. Liquid mixtures of 10 mg were introduced into aluminium DSC crucibles and cooled to −70 °C at a rate of 5 °C/min, kept for 10 min, and then heated at a rate of 5 °C/min. When constructing the liquidus line, only those temperatures at which the complete disappearance of the solid phase was achieved were taken into account.

### 3.4. HES Extraction Experiment

The Fe(III) solution was prepared by dissolving a portion of FeCl_3_ × 6H_2_O accurately weighed on an analytical balance in distilled water. LiCl solutions were prepared by dissolving a sample of salt weighed on an analytical balance in distilled water. The LiCl concentration in solutions was clarified by argentometric titration with a AgNO_3_ solution with a K_2_CrO_4_ indicator.

All extraction experiments were carried out in graduated centrifuge tubes at a temperature of 25 °C and an atmospheric pressure of ~100 kPa. The volume ratio of the organic and aqueous phases V_HES_/V_aq_ was 1/1 (3 mL/3 mL). Fe(III) ions were extracted from a solution of Fe(III) chloride with a concentration of 0.01 mol/L. Extraction was carried out by mixing HES with an aqueous solution of Fe(III) with different concentrations of LiCl and HCl. Stripping was carried out by mixing the Fe(III)-enriched HES phase with distilled water. The tubes were mixed in a shaker IKA Trayster digital (IKA, Cologne, Germany) at 45 rpm for the time required to establish thermodynamic equilibrium. After mixing, the samples were centrifuged at 2500 rpm for 5 min to completely separate the phases on an SM-6MT centrifuge (SIA ELMI, Riga, Latvia). The phases were then separated in separating funnels, and the aqueous phase was analysed.

The concentration of Fe(III) ions was determined spectrophotometrically in the visible region (λ = 420 nm) using sulfosalicylic acid relative to water as an indicator. The concentration of Fe(III) ions in the organic phase after extraction was calculated from the material balance.

The degree of extraction (*E*, %) was determined using Equation (3):

(3) $$E(\%)=\frac{n_{\mathrm{in}}-n_{\mathrm{aq}}}{n_{\mathrm{in}}}\times 100\%,$$

where *n*_in_ and *n*_aq_ are the number of Fe(III) ions in the initial solution and aqueous solution after extraction, respectively.

Extraction data were measured three times, and the values were averaged, with a relative standard deviation of less than 5%.

### 3.5. Born–Oppenheimer Molecular Dynamics

Equimolar mixtures of camphor and thymol with octanol at 298 K were studied using Born–Oppenheimer molecular dynamics. The simulation was performed for systems containing 20 pairs of camphor or thymol and 1-octanol, which included 1080 and 1040 atoms, respectively. The linear dimensions for cubic simulation cells were 42 Å. Periodic boundary conditions were introduced to eliminate the influence of size effects.

The calculations were performed using the CP2K/QUICKSTEP [38] package and the GFN1-xTB [39] package. The density criterion was set to 400 Ry, the target convergence accuracy threshold of the self-consistent field was 1.0 × 10^−6^, and the DIIS minimiser was used. The simulations were performed in the NVT ensemble with a time step of 1 fs. The temperature was controlled using canonical sampling through velocity rescaling thermostat [40]. The system was pre-thermostated for 200 fs with a thermostat time constant of 2, after which the simulation was run for 30 ps with a time constant of 1000.

Trajectories were analysed using the VMD package [41]. In the analysis of the number of hydrogen bonds, threshold values of 3 Å and 20° were set for O···O distances and OHO angles, respectively.

After 25 ps of simulation, the systems were relaxed (Figure S15), and the number of hydrogen bonds formed was analysed (Figure S16, Table S1), revealing that the most common supramolecular structure in both mixtures was the heterodimer. It should be noted that the lower number of corresponding associates for Cam compared to Thy as well as the preference for the heterodimer in which Thy acts as the HBD compared to the complex where it is the HBA are consistent with the results of the free energy calculations of complex formation in the gas phase (Table 2 and Table S9).

### 3.6. Combined DFT+D and DLPNO-CCSD Calculations

The values of the Gibbs energy of complex formation were obtained from the following calculation steps:

1. Geometry was optimised and vibration was calculated for monomers and dimer at the density functional theory level with the PBE0 [42] functional, def2-TZVP [43] basis set, and dispersion interactions taken into account by using the D3(BJ) [44] empirical correction. Calculations were performed using Gaussian 16 C.01 [45]. All structures discussed in this paper correspond to the minima on the potential energy surface.
2. The SP calculation of the electronic energy of the monomers and dimer (for the geometry obtained in the previous step) was performed at the DLPNO-CCSD(T) [46] level of theory with the def2-TZVP basis set. The calculations were performed using Orca 5.0.4 [47] software.

The Gibbs energies of complex formation were calculated according to Equation (4):

(4) $$\Delta G={\Delta G}_{\mathrm{corr}}-{\Delta E}_{e}$$

where Δ*E*_e_ is the electron energy of complexation (formation of an associate from two isolated relaxed molecules), calculated at the DLPNO-CCSD(T) level of theory as the difference in the electron energies of the dimer and monomers; Δ*G*_corr_ is the difference in the thermal corrections to the electron energy obtained from the vibrational calculation for the dimer and monomers.

The choice of the optimal structure from the point of view of the donor–acceptor property for those cases in which this is ambiguous (Hpph, Men, Thy) was made using the example of octanol associates based on a comparison of the Gibbs energy of complexation (at a temperature of 298 K) and total electron energies under the assumption of the same nature of the corresponding complexes for octanol and dodecanol. These energy characteristics are given in Table S17, while the structures of less stable associates are shown in Figure S17. The structures of the associates were visualised using GaussView 6 [48].

The real-space electronic structure analysis was performed for the complexes of octanol within the ‘Atoms in Molecules’ theory [36]. Atomic bonding graphs (Figure 6), atomic charges, and surface integrals for estimations of strength of intermolecular bonding contributions were calculated using the AIMAll program [49].

## 4. Conclusions

In summary, this study demonstrates that the second component in eutectic solvents significantly influences the extraction ability of aliphatic alcohols, as exemplified by Fe(III) ion extraction. Extraction efficiency is determined not only by the energy of hydrogen bonds, which varies depending on whether the second component acts as a hydrogen bond donor (HBD) or acceptor (HBA), but also by the energy of CH···π and CH···O interactions, which are similarly influential. This paper proposes a novel approach to designing eutectic solvents by analysing the total energy of intermolecular interactions between components. This approach is crucial, as it provides an additional method of controlling the extraction capabilities of hydrophobic eutectic solvents, enabling researchers to synthesise effective extractants with tailored properties through the careful selection of components.

## Figures and Tables

**Table 1:** Concentration values (mol/L) of 1-octanol/1-dodecanol for HESs in the ratio aliphatic alcohol:second component 7:3.

	Aliphatic Alcohol	[1-Octanol], mol/L	[1-Dodecanol], mol/L
Second Component			
Camphor		4.41	3.48
2′-Hydroxypropiophenone		4.47	3.64
*L*-Menthol		4.29	3.40
1-Octanoic acid		4.43	3.49
Thymol		4.45	3.53

**Table 2:** Selected energy features of calculated supramolecular associates of 1-octanol (1-dodecanol).

Second Component	Formal Type of the Associate	Δ*G* (kcal/mol) ^(a)^	Bonding Contributions to Intermolecular Interaction Energy (kcal/mol) ^(b)^	
			H-bond	CH···π and CH···O Interactions
Cam	HBA	17.3 (17.3)	12.4	8.2
Hpph	HBA	18.9 (19.1)	14.6	7.9
Men	HBA	20.3 (20.0)	7.9	14.9
OctA	HBD + HBA	21.7 (21.7)	18.2 + 6.4 ^(c)^	-
Thy	HBD	25.0 (25.1)	11.2	15.6

(a) Italic font denotes the Δ*G* value for similar complexes of dodecanol-1, (b) intermolecular interaction energy and its bonding contributions were estimated using the surface integral scheme of electron density analysis [37], (c) the larger value corresponds to the H bond formed by OctA as an HBD.

**Table 3:** Reagents.

Reagent	Supplier	CAS	Purity, wt.% *
1-Octanol	Macklin, Shanghai, China	111-87-5	99.5
1-Dodecanol	Macklin, Shanghai, China	112-53-8	>99
Camphor	Macklin, Shanghai, China	76-22-2	96
2′-Hydroxypropiophenone	Macklin, Shanghai, China	610-99-1	≥98
*L*-Menthol	Acros, Geel, Belgium	2216-51-5	99
1-Octanoic acid	Merck, Darmstadt, Germany	124-07-2	>99
Thymol	Macklin, Shanghai, China	89-83-8	>99
FeCl_3_ × 6H_2_O	Macklin, Shanghai, China	7705-08-0	99
LiCl	Macklin, Shanghai, China	7447-41-8	98
HCl	Macklin, Shanghai, China	7647-01-0	37

* Declared by suppliers.

## Data Availability

The data presented in this study are available in this article and the Supplementary Materials.

## Supplementary Materials

The following supporting information can be downloaded at: https://www.mdpi.com/article/10.3390/molecules29215022/s1; IR spectra; electronic absorption spectra; Born–Oppenheimer molecular dynamics; combined DFT+D and DLPNO-CCSD calculations.
