# Supplementary material for: Design of Eutectic Solvents with Specified Extraction Properties Based on Intermolecular Interaction Energy
Source: Molecules. 2024 Oct 24;29(21):5022. doi: 10.3390/molecules29215022 (PMC11547920; doi:10.3390/molecules29215022)
Supplement: Supplementary file 1 [file molecules-29-05022-s001.zip › molecules-3248172-supplementary.pdf]

SUPPORTING INFORMATION

Design of Eutectic Solvents with Specified Extraction Properties Based on Intermolecular Interaction Energy

Arina V. Kozhevnikova [1], Ekaterina S. Uvarova [1], Varvara E. Maltseva [1], Ivan V. Ananyev [1], Nikita A. Milevskii [1], Igor S. Fedulov [2], Yulia A. Zakhodyaeva [1] and Andrey A. Voshkin [1,*]

[1] Kurnakov Institute of General and Inorganic Chemistry, Russian Academy of Sciences, 119991 Moscow, Russia; ak@igic.ras.ru (A.V.K.); uvarovacatya@yandex.ru (E.S.U.); varyamalceva@mail.ru (V.E.M.); i.ananyev@gmail.com (I.V.A.); mna@igic.ras.ru (N.A.M.); yz@igic.ras.ru (Y.A.Z.)
[2] Institute for African Studies, Russian Academy of Sciences, 123001 Moscow, Russia; if345@ya.ru
* Correspondence: aav@igic.ras.ru; Tel.: +7-495-775-65-81

Table of Contents

IR spectra **2**

Electronic absorption spectra **7**

Born-Oppenheimer molecular dynamics **9**

Combined DFT+D and DLPNO-CCSD calculations **10**

IR spectra

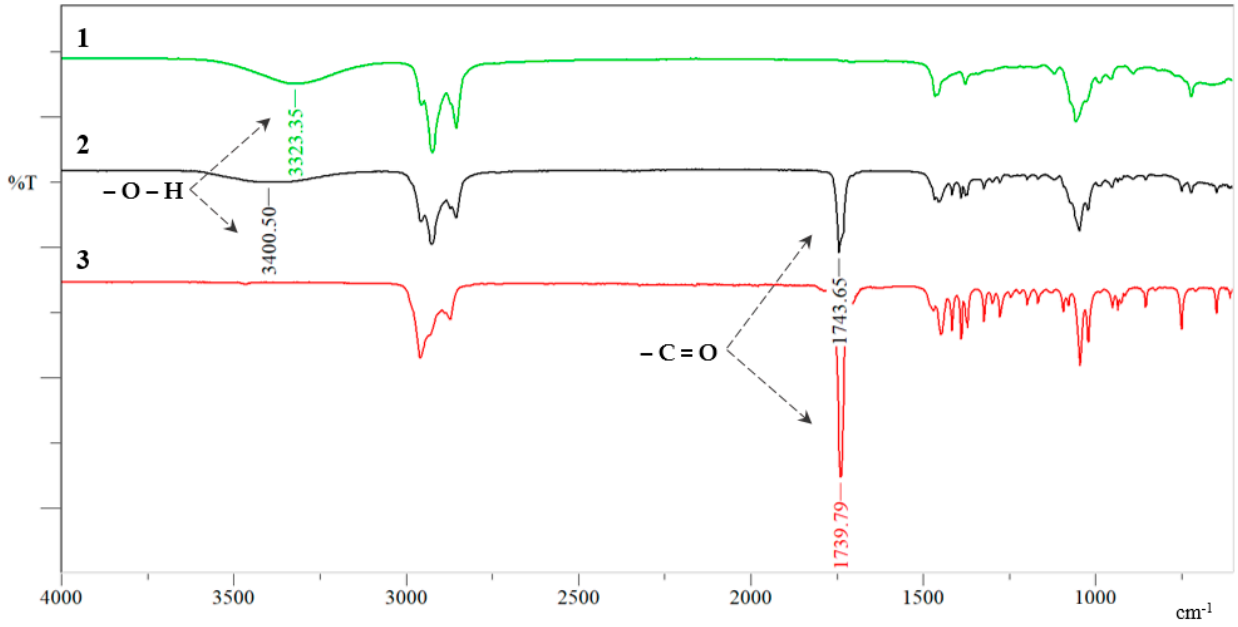

Figure S1. IR spectra of 1-octanol – **1**, HES 1-octanol/camphor – **2**, camphor – **3**.

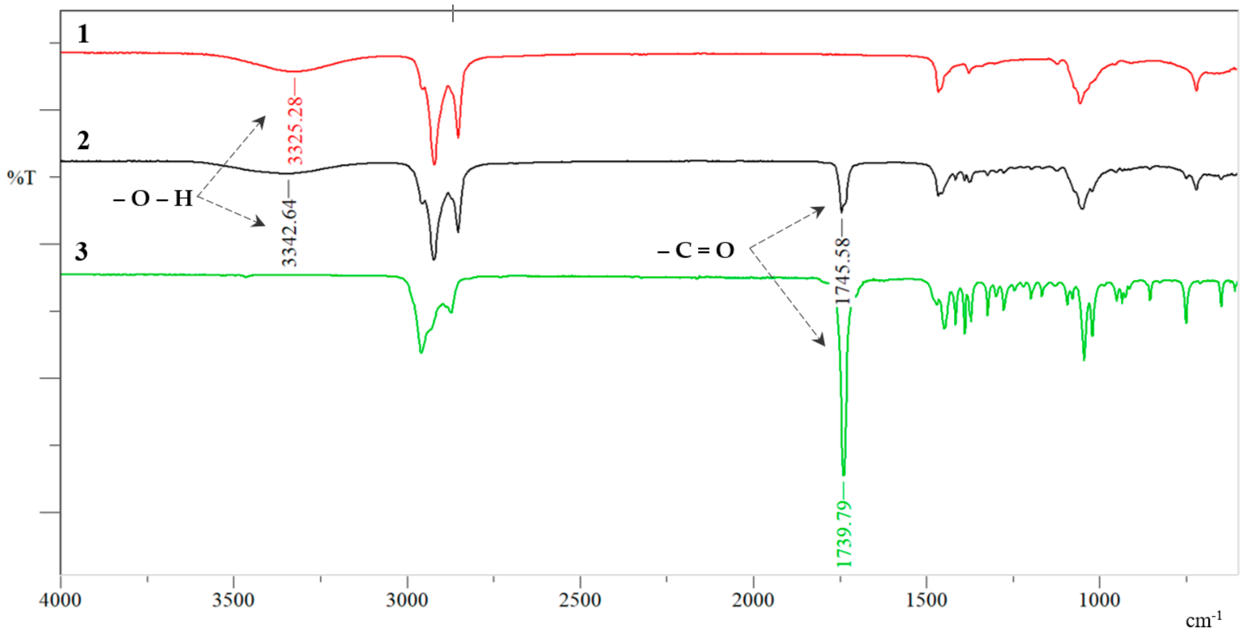

Figure S2. IR spectra of 1-dodecanol – **1**, HES 1- dodecanol/camphor – **2**, camphor – **3**.

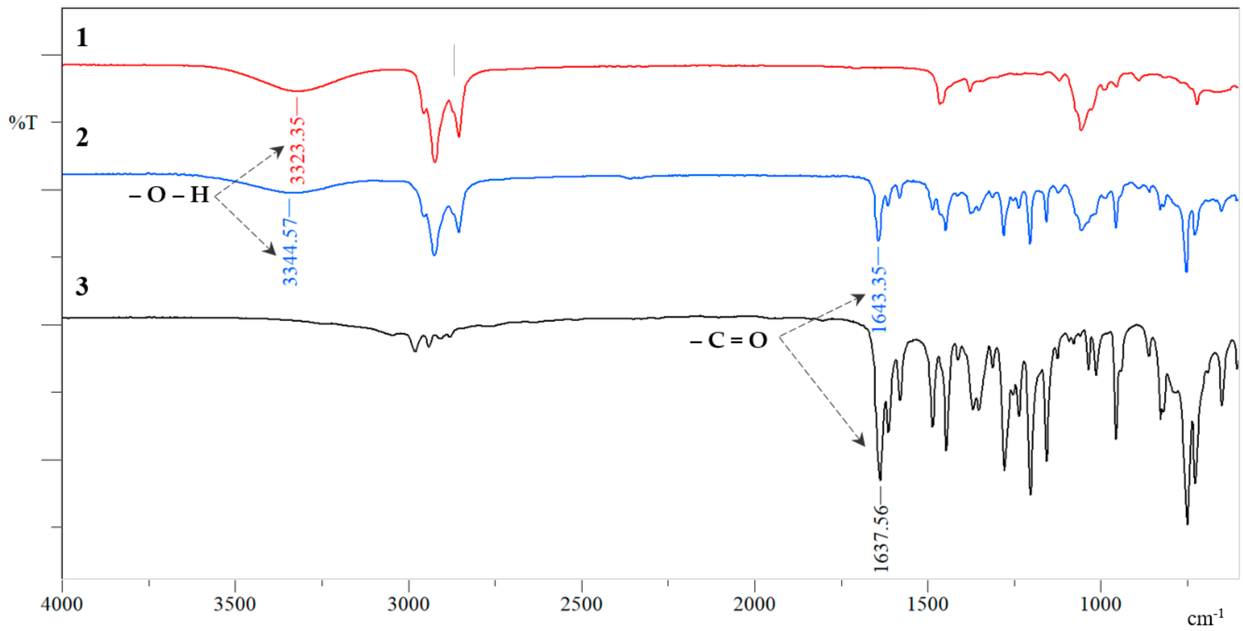

Figure S3. IR spectra of 1-octanol – **1**, HES 1-octanol/2′-hydroxypropiophenone – **2**, 2′-hydroxypropiophenone – **3**.

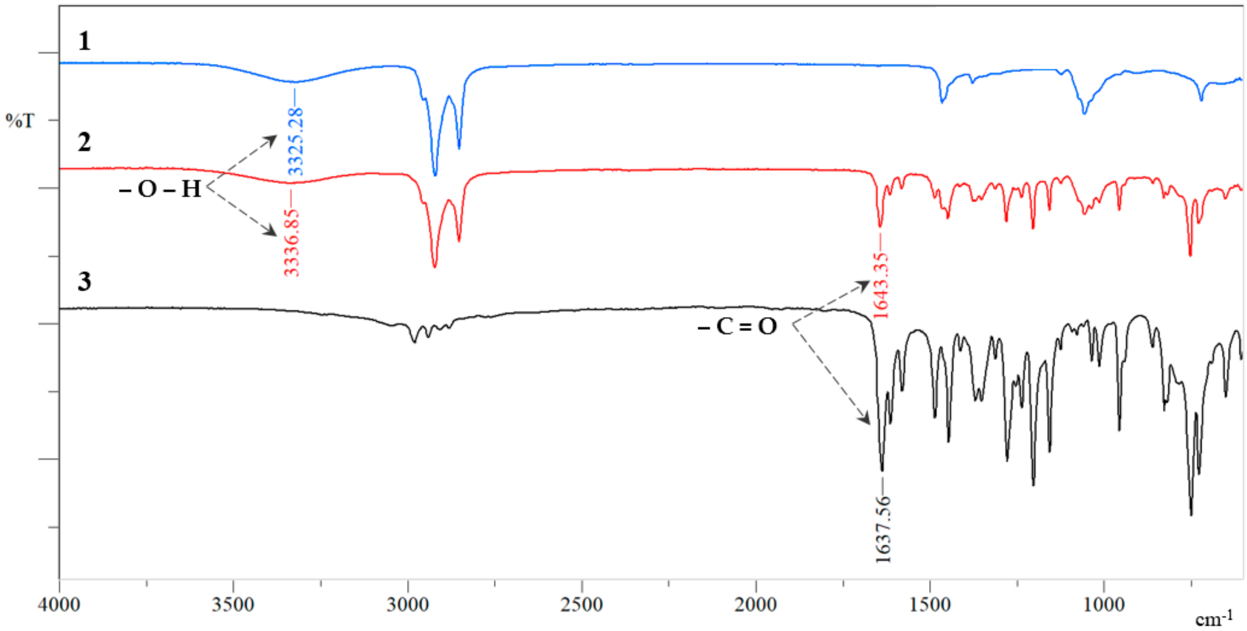

Figure S4. IR spectra of 1-dodecanol – **1**, HES 1-dodecanol/2′-hydroxypropiophenone – **2**, 2′-hydroxypropiophenone – **3**.

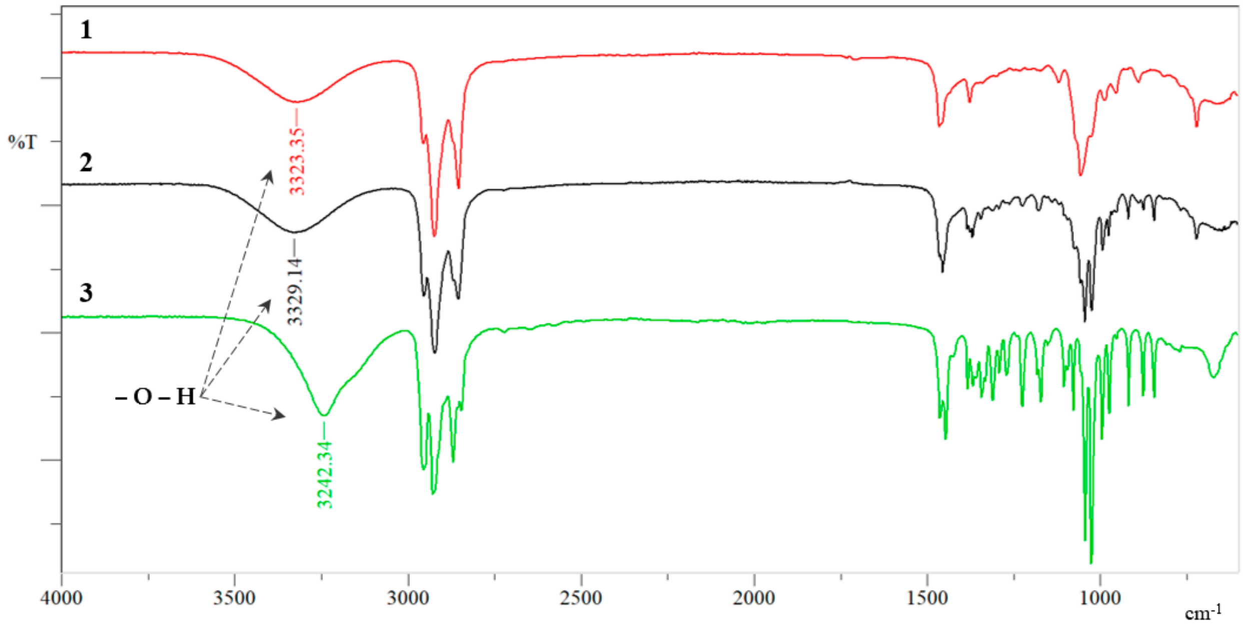

Figure S5. IR spectra of 1-octanol – **1**, HES 1-octanol/menthol – **2**, menthol – **3**.

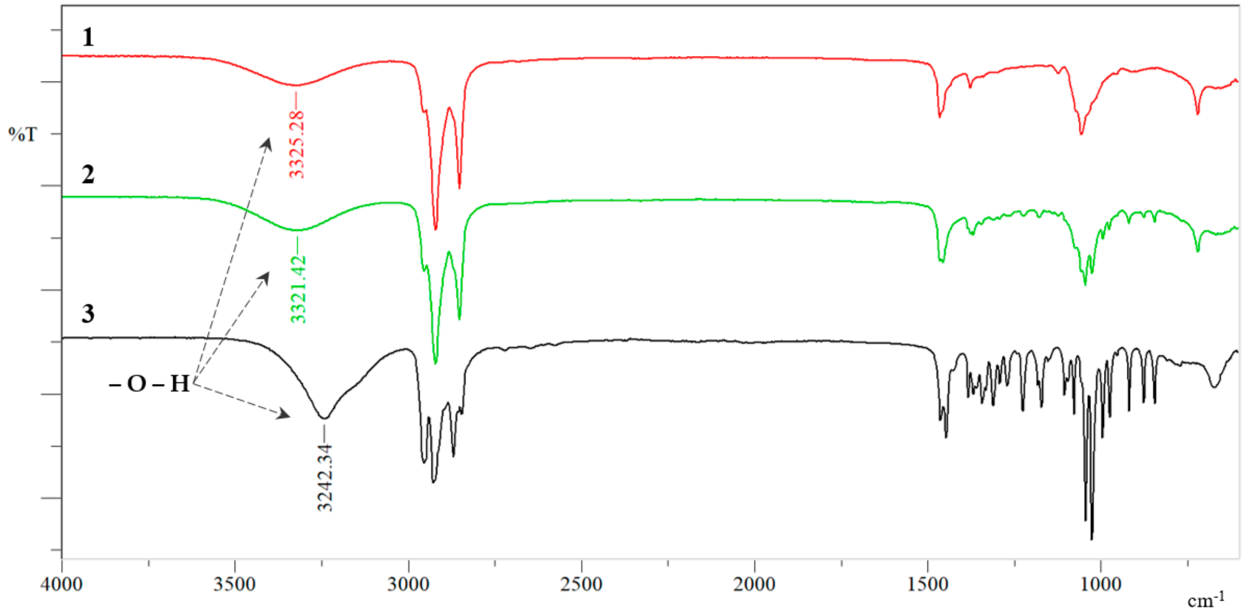

Figure S6. IR spectra of 1-dodecanol – **1**, HES 1- dodecanol/menthol – **2**, menthol – **3**.

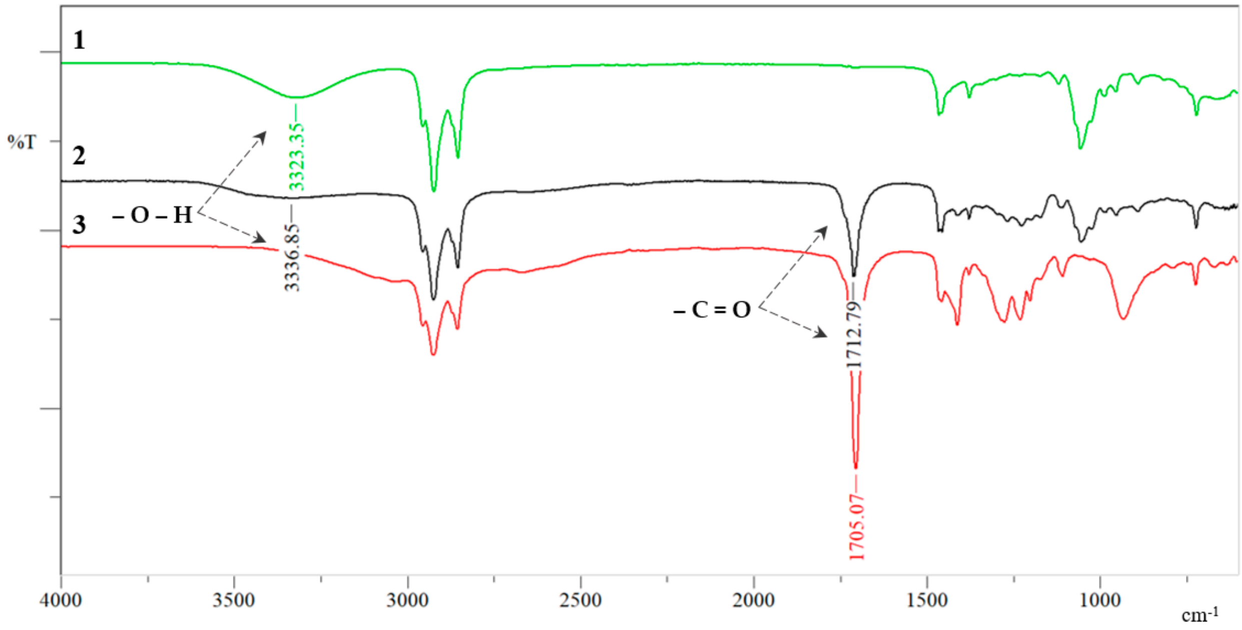

Figure S7. IR spectra of 1-octanol – **1**, HES 1-octanol/1-octanoic acid – **2**, 1-octanoic acid – **3**.

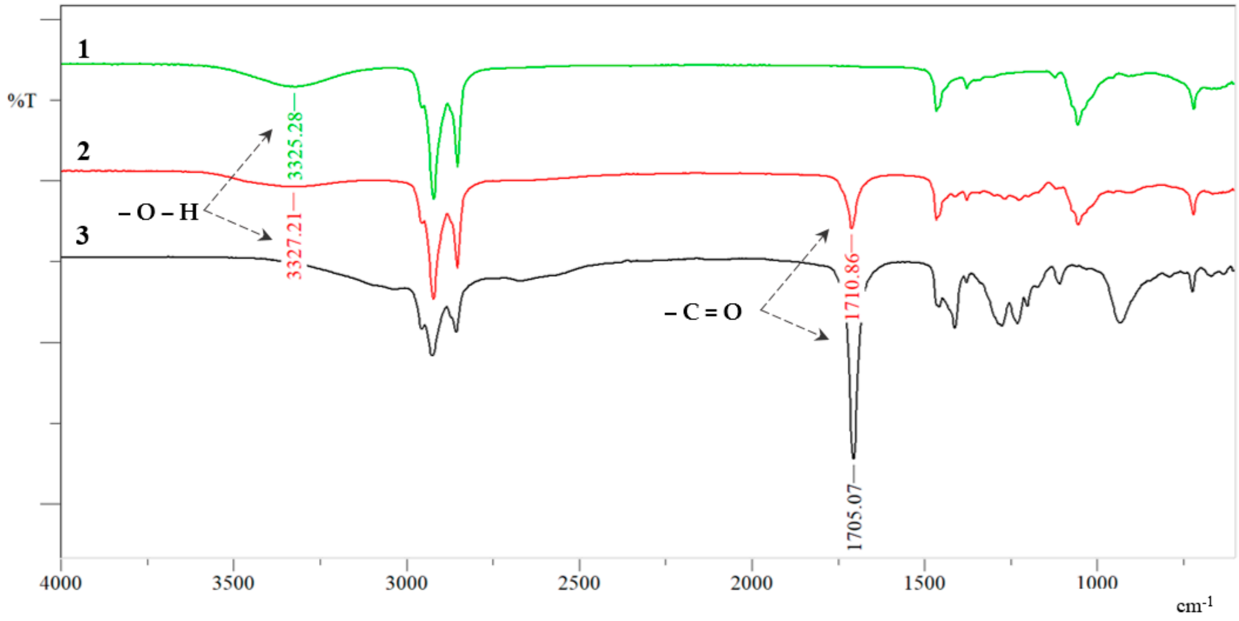

Figure S8. IR spectra of 1-dodecanol – **1**, HES 1- dodecanol/1-octanoic acid – **2**, 1-octanoic acid – **3**.

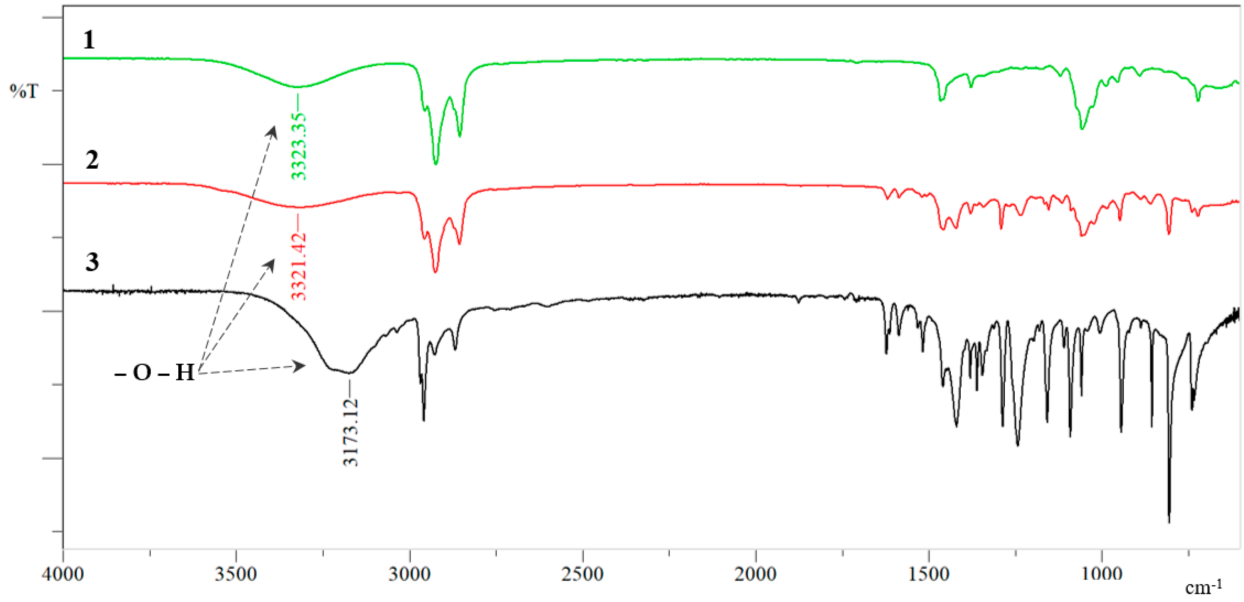

Figure S9. IR spectra of 1-octanol – **1**, HES 1-octanol/thymol – **2**, thymol – **3**.

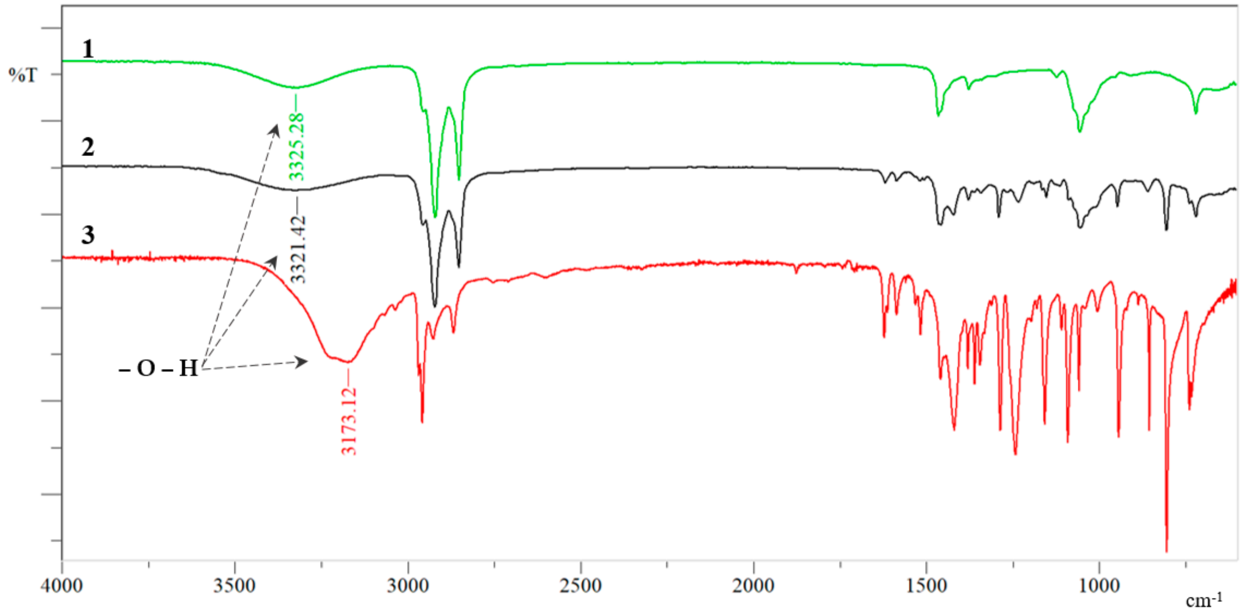

Figure S10. IR spectra of 1-dodecanol – **1**, HES 1- dodecanol/thymol – **2**, thymol – **3**.

Electronic absorption spectra

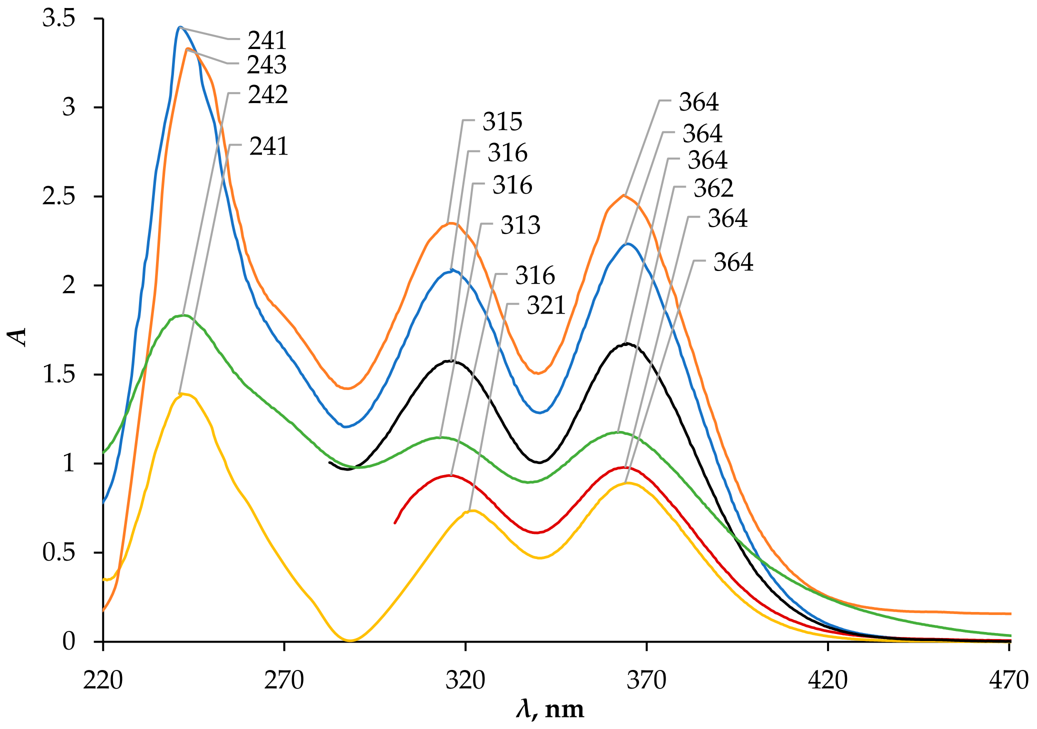

Figure S11. Electronic absorption spectra of HESs based on 1-octanol after extraction in HCl: yellow line – Oct/Cam, blue line – Oct/Men, orange line – Oct/OctA, red line – Oct/Thy, black line – Oct in toluene, green line – $H[FeCl_4]$ (0.003 mol/L $FeCl_3$ in 8 mol/L HCl).

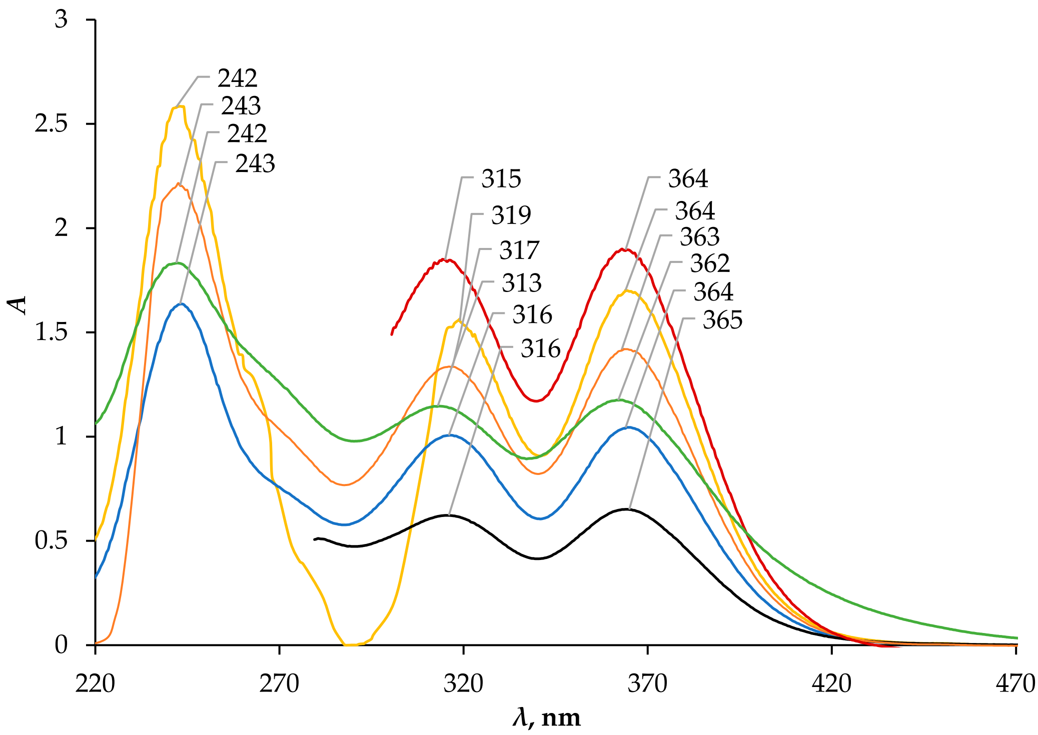

Figure S12. Electronic absorption spectra of HESs based on 1-dodecanol after extraction in HCl: yellow line – Dod/Cam, blue line – Dod/Men, orange line – Dod/OctA, red line – Dod/Thy, black line – Dod in toluene, green line – $H[FeCl_4]$ (0.003 mol/L $FeCl_3$ in 8 mol/L HCl).

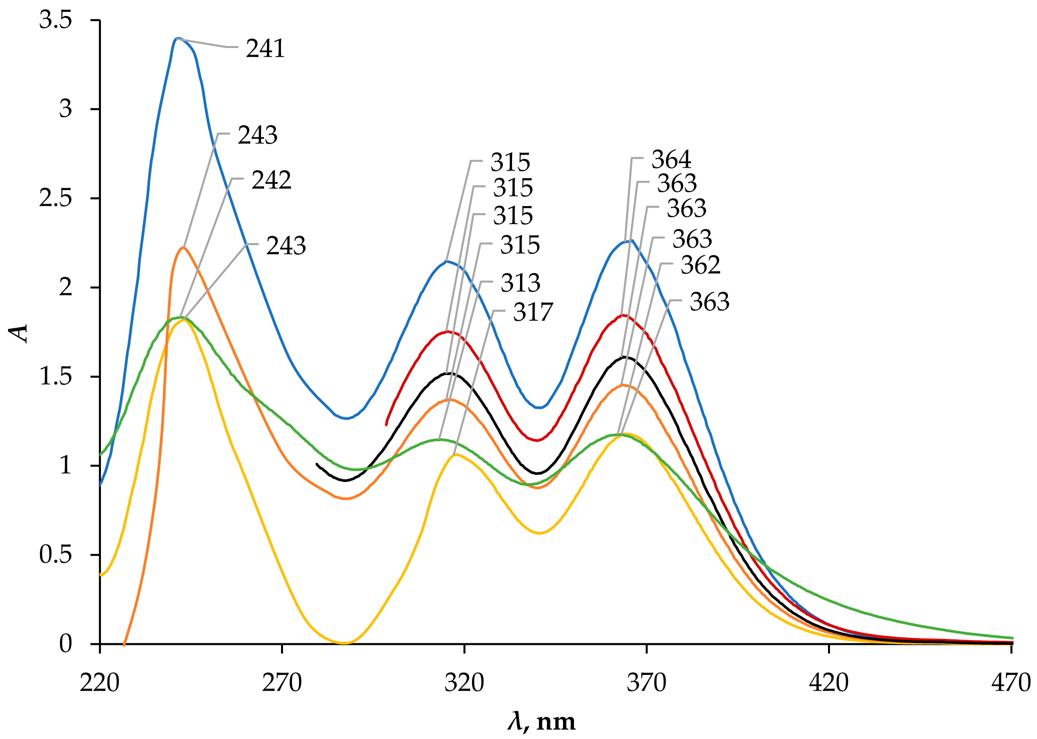

Figure S13. Electronic absorption spectra of HESs based on 1-octanol after extraction in LiCl: yellow line – Oct/Cam, blue line – Oct/Men, orange line – Oct/OctA, red line – Oct/Thy, black line – Oct in toluene, green line – H[$FeCl_4$] (0.003 mol/L $FeCl_3$ in 8 mol/L HCl).

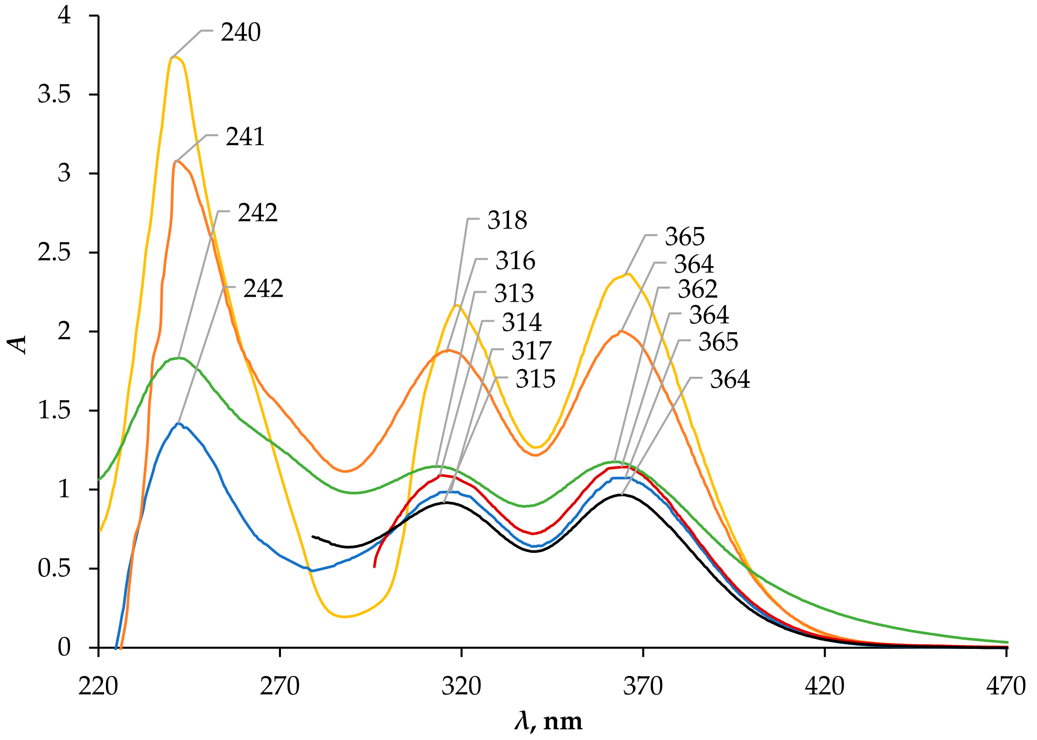

Figure S14. Electronic absorption spectra of HESs based on 1-dodecanol after extraction in LiCl: yellow line – Dod/Cam, blue line – Dod/Men, orange line – Dod/OctA, red line – Dod/Thy, black line – Dod in toluene, green line – H[$FeCl_4$] (0.003 mol/L $FeCl_3$ in 8 mol/L HCl).

Born-Oppenheimer molecular dynamics

Table S1. The average number of selected types of hydrogen bonds.

Formal type of the associate (Cam)	N_{HB}	Formal type of the associate (Thy)	N_{HB}
**Cam (HBA)/Oct (HBD)**	**1.87**	**Thy (HBD)/Oct (HBA)**	**1.96**
Oct/Oct	1.11	Thy (HBA)/Oct (HBD)	0.60
		Thy/Thy	0.85
		Oct/Oct	0.37

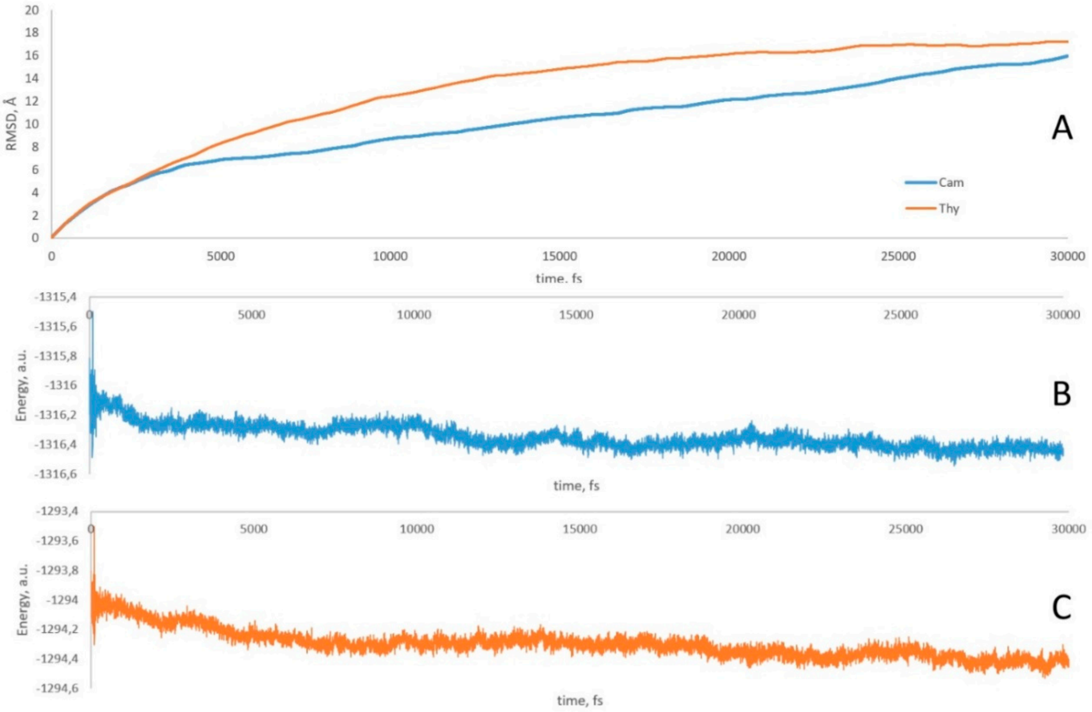

Figure S15. Dependence of RMSD (A) and total energy of the system (B – Cam, C – Thy) on the simulation time.

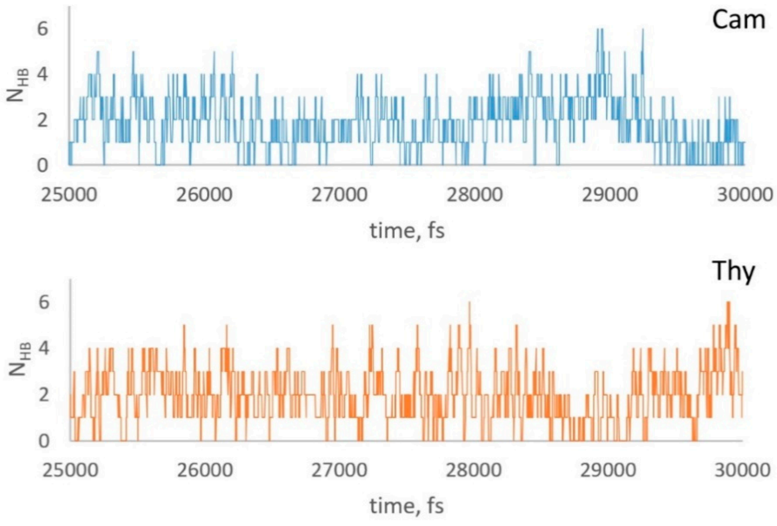

Figure S16. Dependence of the number of hydrogen bonds of the “Cam/Oct” and “Thy (HBD)/Oct” types on the simulation time.

Combined DFT+D and DLPNO-CCSD calculations

Below are the coordinates of the atomic nuclei of octanol (Tables S2-S10) and dodecanol (Tables S11-S16) associates and the values of the total electron energy calculated at the theoretical level DLPNO-CCSD(T)/def2-TZVP.

Table S2. Cam/Oct. E = -855.268765453 a.u.

O	-2.90950200	-2.04710900	0.45365500
C	-2.81055400	1.36763000	-0.09712100
C	-3.46235200	0.29462100	0.82524300
C	-3.60385000	0.92652400	-1.35263100
C	-4.97463600	0.53667700	0.58136300
C	-5.05940200	1.02027800	-0.88296200
C	-3.26957200	-0.56253500	-1.44579000
C	-3.17702300	-0.95347500	0.01690100
C	-3.09123000	2.79767000	0.33682600
C	-1.30141700	1.20053900	-0.23406600
C	-3.04708000	0.21429800	2.26789300
H	-3.38152400	1.49326200	-2.25808200
H	-5.33184400	1.29229500	1.28404400
H	-5.55765200	-0.36755300	0.76893300
H	-5.71669600	0.39688800	-1.49193600
H	-5.43890600	2.04103200	-0.94584400
H	-2.31174100	-0.77589200	-1.92588100
H	-4.02928600	-1.15975000	-1.95596800
H	-4.14735600	3.00065400	0.51229600
H	-2.74219500	3.49468100	-0.43020500
H	-2.55119300	3.03027900	1.25790700
H	-0.91529200	1.87552600	-1.00265800
H	-0.99558000	0.18761700	-0.50131300
H	-0.80371200	1.45770700	0.70431500
H	-3.30812600	1.13046600	2.80259300
H	-3.54311000	-0.62359000	2.76197600
H	-1.97104500	0.05285800	2.36200400
C	7.57383500	2.13652600	-0.62774700
C	6.26084000	1.53694500	-0.15522000
H	7.44287700	3.15718300	-0.99448200
H	8.30770300	2.16522900	0.18271400
H	8.00565900	1.54468300	-1.43981400

C	6.41797500	0.11492700	0.36158600
H	5.83489200	2.16094300	0.63973600
H	5.53763900	1.55655000	-0.97659900
C	5.14167000	-0.49932000	0.91973900
H	6.80333300	-0.52078100	-0.44582200
H	7.18777700	0.10963200	1.14212600
H	5.38206400	-1.46530500	1.37855800
H	4.76082900	0.13351800	1.73151300
C	4.04161200	-0.70936800	-0.10857100
C	2.81214300	-1.39300800	0.46537400
H	4.43405200	-1.30776900	-0.94051800
H	3.74335500	0.25171800	-0.54153500
C	1.70942400	-1.59888400	-0.55776100
H	3.10279600	-2.36070500	0.89260200
H	2.42772900	-0.79708700	1.30293500
C	0.48685800	-2.26656500	0.02600800
H	1.41205000	-0.63485100	-0.98583100
H	2.07710600	-2.20733300	-1.39113900
H	0.76518700	-3.24181100	0.45221000
H	0.09172200	-1.65716300	0.85221000
O	-0.48180300	-2.42214400	-0.98734200
H	-1.32452400	-2.63999100	-0.56593500

Table S3. Hpph (HBD)/Oct. E = -888.759337560248 a.u.

C	-2.87653700	-1.40077100	2.04346300
C	-2.93958100	-0.90060000	0.75898400
C	-1.89628300	-1.10911400	-0.14614300
C	-0.76629800	-1.83800800	0.25557000
C	-0.73586600	-2.33596100	1.56143400
C	-1.76606300	-2.12951900	2.45382700
H	-3.70090900	-1.22694400	2.72626300
H	-3.80693900	-0.34022700	0.42751700
H	0.15078500	-2.88801600	1.84884200
H	-1.71016200	-2.52989600	3.45856100
C	0.45227400	-2.10097800	-0.57420200
C	0.47968000	-1.73550400	-2.03520400
C	1.77718600	-2.11879400	-2.71265700
H	-0.38731200	-2.19661500	-2.51953200

H	0.28391500	-0.66240700	-2.11649700
H	1.75381600	-1.83708500	-3.76728200
H	2.62691400	-1.62280600	-2.24051100
H	1.95691300	-3.19269900	-2.64456200
O	-1.98000800	-0.60247900	-1.39041100
H	-2.54972000	0.19263200	-1.38013700
O	1.42081200	-2.61671800	-0.05625300
C	5.62064500	0.84749300	0.85589300
C	4.17428200	0.47656600	0.58122000
H	6.26772200	-0.03255900	0.85786000
H	6.00150600	1.53859100	0.09824800
H	5.72385300	1.33794500	1.82821100
C	3.24306200	1.67766600	0.56686400
H	4.10188400	-0.04480500	-0.37997100
H	3.82471000	-0.24107500	1.33145800
C	1.79594100	1.30055300	0.30869000
H	3.31771800	2.20789000	1.52487600
H	3.57889400	2.38925700	-0.19843900
H	1.73526300	0.74387100	-0.63423300
H	1.45865000	0.60335200	1.08504600
C	0.84306300	2.48268900	0.24579600
C	-0.59390600	2.03811400	0.03995400
H	1.14418800	3.15254500	-0.56944200
H	0.92149000	3.07120600	1.16846000
C	-1.58371000	3.17693500	-0.13116100
H	-0.63607700	1.39112700	-0.83974200
H	-0.90050400	1.41544200	0.88891200
C	-3.02528200	2.72752500	-0.17679100
H	-1.49184900	3.87846500	0.70654300
H	-1.35585700	3.74235900	-1.04118800
H	-3.68467100	3.59702300	-0.26559700
H	-3.28100400	2.19662500	0.74901000
O	-3.23935800	1.85978500	-1.29685800
H	-4.17042400	1.88234400	-1.52772100

Table S4. Hpph (HBA, =O)/Oct. E = -888.754017398847 a.u.

C	0.54849900	-2.82624800	-0.03945100
C	1.86068000	-3.13424700	-0.33881900

C	2.85375200	-2.16057800	-0.27118700
C	2.52890900	-0.84614500	0.09897600
C	1.18919500	-0.56720600	0.39195200
C	0.20608400	-1.53225200	0.32956200
H	-0.20827700	-3.60031900	-0.09617600
H	2.13175900	-4.14458700	-0.63235200
H	0.93340400	0.44595600	0.67777200
H	-0.81976200	-1.27801200	0.56414300
C	3.49376100	0.28718400	0.20882400
C	4.97636900	0.05018500	0.11038300
C	5.79506400	1.29423400	0.37936400
H	5.23902100	-0.76558200	0.79105200
H	5.18095100	-0.35686200	-0.88518700
H	6.86018000	1.07126200	0.29366600
H	5.54996100	2.08786200	-0.32729200
H	5.60386600	1.68406200	1.38029700
O	4.13257600	-2.48446500	-0.57590700
H	4.16700000	-3.41573200	-0.81330700
O	3.08106700	1.41710300	0.39434400
C	-7.42481800	-1.50910200	0.29407100
C	-5.91018800	-1.40419300	0.32814900
H	-7.76600200	-2.51508600	0.54910300
H	-7.88279900	-0.81314700	1.00255000
H	-7.81323000	-1.26803100	-0.69948800
C	-5.40084100	-0.01605700	-0.02233600
H	-5.54407200	-1.68032300	1.32391000
H	-5.47472000	-2.13252900	-0.36609200
C	-3.88693000	0.09993700	0.00931200
H	-5.76567800	0.26234400	-1.01907000
H	-5.83528400	0.71463100	0.67137400
H	-3.52413300	-0.17724900	1.00750800
H	-3.45348600	-0.63241300	-0.68418800
C	-3.37171700	1.48565400	-0.34115900
C	-1.85720600	1.58138200	-0.30638900
H	-3.79928100	2.21834600	0.35434300
H	-3.73599800	1.76667400	-1.33743300
C	-1.31861700	2.95803200	-0.65318500
H	-1.49774400	1.30667500	0.69061000

H	-1.43224900	0.84566700	-1.00136400
C	0.19222700	3.02907100	-0.60428000
H	-1.64971800	3.24821600	-1.65717900
H	-1.72638900	3.70189000	0.04079100
H	0.53007700	4.01205800	-0.96247000
H	0.61557900	2.27289900	-1.28217300
O	0.62795400	2.80738900	0.71788400
H	1.53891200	2.48320100	0.68336500

Table S5. Hpph (HBA, -OH)/Oct. E = -888.749956296477 a.u.

C	5.66518400	0.52195200	-1.01887500
C	5.00019900	-0.46787900	-0.32193500
C	3.72537800	-0.24278300	0.18603100
C	3.09328600	0.99126700	0.00067200
C	3.78847800	1.96576300	-0.71984500
C	5.05398700	1.75116100	-1.22466400
H	6.65897900	0.32833300	-1.40550700
H	5.46619200	-1.43814300	-0.17388000
H	3.28264300	2.91301600	-0.86131200
H	5.56367100	2.53348000	-1.77307100
C	1.74047900	1.39871700	0.51434300
C	0.98918000	0.52100300	1.47863700
C	-0.24681700	1.18943300	2.04095900
H	0.72505200	-0.39378400	0.93814900
H	1.67456200	0.19787100	2.26767100
H	-0.75947000	0.51766100	2.73224500
H	0.00809000	2.10610600	2.57539400
H	-0.94207400	1.45990300	1.24591800
O	3.08741300	-1.25127900	0.85102800
H	3.71566900	-1.96712100	0.98938100
O	1.27526200	2.45737400	0.15519600
C	-7.32475600	1.83428300	-0.57999700
C	-5.87154200	1.63811800	-0.18528900
H	-7.66762100	2.84919500	-0.36599900
H	-7.46963700	1.65292500	-1.64878600
H	-7.97624500	1.14210100	-0.03890500
C	-5.36148700	0.23519600	-0.46985700
H	-5.24389800	2.36302800	-0.71622200

H	-5.74650800	1.85650000	0.88165000
C	-3.90851500	0.02849900	-0.07969500
H	-5.98814900	-0.49239300	0.06157500
H	-5.48556900	0.01495700	-1.53770200
H	-3.28229800	0.75505500	-0.61208800
H	-3.78418200	0.25122500	0.98758700
C	-3.39675700	-1.37382700	-0.36216300
C	-1.94250600	-1.56526200	0.02855800
H	-3.51778800	-1.59640400	-1.42955200
H	-4.02049900	-2.10280400	0.17084900
C	-1.41544600	-2.96326200	-0.24315900
H	-1.32248700	-0.84217500	-0.51048300
H	-1.82359700	-1.33751700	1.09568800
C	0.04415600	-3.12430800	0.11895100
H	-1.99837100	-3.70163100	0.31952000
H	-1.53797000	-3.20816900	-1.30428500
H	0.34169700	-4.17736300	0.01562100
H	0.19194800	-2.84298500	1.17204400
O	0.82608100	-2.30746700	-0.72937600
H	1.63282000	-2.05832000	-0.26630700

Table S6. Men (HBA)/Oct. E = -857.672334332875 a.u.

C	-2.07960000	0.51326100	0.40577400
C	-2.14039300	0.98622900	-1.03872700
C	-1.62023100	1.67221400	1.28639500
C	-0.31379200	2.68941300	-0.61507100
C	-0.80949200	1.55370400	-1.50043700
C	-0.27864800	2.22667100	0.83507800
C	-3.37965600	-0.16165700	0.87215600
C	1.03987400	3.19824300	-1.07855900
C	-3.23128200	-0.74707100	2.27027000
C	-4.60157300	0.74401500	0.80040000
H	-1.29571100	-0.25515400	0.44793200
H	-2.90297200	1.77587400	-1.11396200
H	-1.55183900	1.34055300	2.32468900
H	-2.36769600	2.47402500	1.26444900
H	-1.03701400	3.51419000	-0.68431000
H	-0.89871700	1.90074400	-2.53782800

H	-0.06777700	0.74653700	-1.50063400
H	0.02722700	3.05540600	1.48140100
H	0.49043200	1.44972400	0.93952000
H	-3.55244600	-0.99677200	0.18628900
H	1.00395100	3.54752800	-2.11367000
H	1.38595300	4.02671300	-0.45555000
H	1.79001800	2.40313900	-1.02129600
H	-3.18506600	0.03332400	3.03458300
H	-4.08418700	-1.38623000	2.50989600
H	-2.32621800	-1.35493500	2.35690900
H	-4.49761100	1.61605000	1.45218300
H	-5.49187700	0.20037700	1.12549300
H	-4.79015700	1.09753200	-0.21523600
C	6.42453300	0.62009400	-0.20909700
C	5.30518900	-0.38752900	-0.01329700
H	7.17088200	0.26090800	-0.92125400
H	6.03542700	1.57011400	-0.58653000
H	6.93710500	0.82772500	0.73446700
C	4.25246300	0.09095300	0.97452400
H	4.83782400	-0.60168300	-0.97965300
H	5.72134800	-1.33855000	0.34009100
C	3.14539200	-0.91506200	1.25667000
H	4.74783600	0.35228900	1.91685900
H	3.80633700	1.02215800	0.60104400
H	2.49831700	-0.51728400	2.04742100
H	3.58297500	-1.83663900	1.66019900
C	2.28481600	-1.25079500	0.04958100
C	1.08432100	-2.11908700	0.38622900
H	2.88568000	-1.74756300	-0.71942300
H	1.92992600	-0.31582500	-0.40422900
C	0.17351700	-2.35706700	-0.80517400
H	1.42378800	-3.07537400	0.80212300
H	0.50938200	-1.63314400	1.18577800
C	-1.12510500	-3.05949000	-0.44799200
H	-0.08524500	-1.39112800	-1.25227000
H	0.69483800	-2.92183500	-1.58503500
H	-0.94532300	-4.11789300	-0.23795600
H	-1.53051500	-2.61965300	0.47638100

O	-2.07558600	-3.00828700	-1.48327200
H	-2.27181700	-2.07397500	-1.64374300
O	-2.53621200	-0.11323100	-1.85568300
H	-2.48466700	0.15862800	-2.77582100

Table S7. Men (HBD)/Oct. E = -857.67115223776 a.u.

O	-3.40361100	-0.51466900	-1.23319000
C	-2.07055200	0.15386200	0.70902300
C	-2.90239100	0.58354500	-0.50281900
C	-1.55987600	1.38706000	1.44792100
C	-1.56792300	2.74348400	-0.67201600
C	-2.11901700	1.52689900	-1.40314200
C	-0.75461200	2.29708200	0.53516700
C	-2.80780800	-0.85565600	1.60056000
C	-0.75814700	3.62964200	-1.60212500
C	-1.86172100	-1.53483300	2.58100200
C	-4.00083400	-0.26181800	2.33760100
H	-1.18888900	-0.36888300	0.30893400
H	-3.79045100	1.11512200	-0.13904000
H	-0.95340600	1.08737200	2.30714500
H	-2.40983900	1.95068900	1.85012600
H	-2.42200500	3.32577800	-0.29777400
H	-2.76189500	1.83430100	-2.23352400
H	-1.28225900	0.97040500	-1.84808200
H	-0.39611600	3.17148700	1.08818000
H	0.13906000	1.76421300	0.18222800
H	-3.19373500	-1.62216800	0.92027200
H	-1.35528700	3.96400900	-2.45427900
H	-0.38528400	4.51639100	-1.08270700
H	0.10657200	3.08585200	-1.99566400
H	-1.46767700	-0.83000900	3.31862700
H	-2.37549100	-2.32658700	3.13236100
H	-1.00907100	-1.98720500	2.06618200
H	-3.68799300	0.48252100	3.07511600
H	-4.54214800	-1.04428500	2.87530000
H	-4.70757000	0.21377300	1.65456900
H	-2.67031600	-0.94290900	-1.70257800
C	6.65918500	1.25491100	0.19319100
C	5.60145200	0.18909800	-0.03440300
H	7.38116600	1.28824800	-0.62583000
H	6.20538000	2.24649200	0.27659000
H	7.21259800	1.06891900	1.11799400
C	4.58391200	0.12387500	1.09431800
H	5.09224200	0.38182200	-0.98404300

H	6.08165000	-0.79115800	-0.14027600
C	3.54951100	-0.98344400	0.95067000
H	5.11928100	-0.00942700	2.04151000
H	4.07006500	1.09054400	1.17217100
H	2.93907900	-1.02014300	1.86038400
H	4.06061700	-1.95256400	0.89006400
C	2.62609100	-0.82727900	-0.24678800
C	1.52915000	-1.87699100	-0.29166800
H	3.20222000	-0.86924800	-1.17728000
H	2.16637500	0.16908300	-0.21880700
C	0.58867500	-1.69674100	-1.46971000
H	1.97930900	-2.87680000	-0.32068100
H	0.95427500	-1.83054900	0.64146900
C	-0.56085400	-2.67430100	-1.45589800
H	0.17719300	-0.68234100	-1.45283200
H	1.13264200	-1.79230900	-2.41479100
H	-0.19432200	-3.69374200	-1.62959500
H	-1.05369400	-2.65970700	-0.47415900
O	-1.48184300	-2.29799200	-2.47182300
H	-2.13408500	-2.99607400	-2.57248700

Table S8. OctA/Oct. E = -854.353550775473 a.u.

O	0.72891800	-0.77546400	1.41432400
O	1.62471600	-1.44421800	-0.52181400
C	6.56700300	0.28813400	-0.37264000
C	5.39720700	0.36147200	0.59344000
C	7.82804000	0.95059500	0.15433700
C	4.14077800	-0.30522300	0.06081300
C	8.99925500	0.87806200	-0.81114500
C	2.98473400	-0.22278700	1.03579800
C	10.25450900	1.54255200	-0.27365800
C	1.72642200	-0.87801300	0.54809100
H	6.77857400	-0.76274500	-0.60547400
H	6.28121500	0.75494700	-1.32332800
H	5.68362400	-0.10419000	1.54489000
H	5.18457400	1.41271700	0.82556100
H	7.61634800	2.00174400	0.38780800
H	8.11381000	0.48345400	1.10528600
H	3.84784600	0.15490100	-0.88784500
H	4.34444000	-1.35513200	-0.17011600
H	9.20880500	-0.17242300	-1.04366900
H	8.71221200	1.34526400	-1.76028200
H	2.73831000	0.81697500	1.27758900
H	3.23888900	-0.68651600	1.99549300

H	10.07967600	2.60146800	-0.06327100
H	11.08124700	1.47734400	-0.98459700
H	10.57988400	1.07239800	0.65864300
H	-0.06171000	-1.23526700	1.02225800
C	-10.23744300	1.69423400	0.44173900
C	-9.00242400	1.50106500	-0.42054900
H	-11.02025900	2.23871400	-0.09113400
H	-10.65430400	0.73212900	0.75265000
H	-9.99959000	2.25693200	1.34887200
C	-7.89232300	0.74585700	0.29110900
H	-9.27292300	0.96344100	-1.33674400
H	-8.62281000	2.47698600	-0.74474200
C	-6.65165900	0.54631200	-0.56211700
H	-7.61986000	1.28276000	1.20849100
H	-8.27048100	-0.23150200	0.61642600
H	-6.92478800	0.00934300	-1.47912600
H	-6.27397500	1.52387200	-0.88708000
C	-5.54320900	-0.20877800	0.15072900
C	-4.30309600	-0.40793800	-0.70401800
H	-5.91987900	-1.18644600	0.47543100
H	-5.26908600	0.32788000	1.06729400
C	-3.19940900	-1.16499200	0.01408300
H	-4.57875200	-0.94434900	-1.62032000
H	-3.92711500	0.57052400	-1.02773800
C	-1.97291300	-1.35260800	-0.84509400
H	-2.91499400	-0.63098200	0.92782600
H	-3.56123000	-2.14980100	0.32795900
H	-2.23701300	-1.89350000	-1.76262500
H	-1.56705000	-0.37602500	-1.14280600
O	-1.00327500	-2.08966900	-0.11701400
H	-0.16933800	-2.10777200	-0.62105700

Table S9. Thy (HBD)/Oct. E = -854.068808926419 a.u.

O	-2.34392300	-0.06756300	1.67894800
C	-2.93519000	1.06332300	-0.92554600
C	-1.61335000	1.24691500	-0.21660400
C	-2.86111000	1.29133800	-2.42715900
C	-4.00211400	1.95980700	-0.29542300
C	-1.40671300	0.68993400	1.05320900
C	-0.57112000	1.99328500	-0.75413700
C	0.80820400	1.65948300	1.19299200
C	-0.21762300	0.89983000	1.73884100
C	0.61819200	2.20065700	-0.07291600
C	2.07831300	1.89140500	1.95293000

H	-3.25400100	0.02223800	-0.78878600
H	-2.65166100	2.33740500	-2.66613000
H	-3.81793900	1.03893000	-2.88933000
H	-2.08715800	0.67492800	-2.89020600
H	-4.08569000	1.78457700	0.77860000
H	-4.97939300	1.77955000	-0.75130000
H	-3.74538500	3.01199800	-0.44445400
H	-0.68747100	2.42551200	-1.74073900
H	-0.10660200	0.43866800	2.71452300
H	1.40719800	2.78525600	-0.53378500
H	1.99639100	2.77713500	2.59055500
H	2.31642700	1.04507200	2.60020800
H	2.92039600	2.05130000	1.27685200
H	-2.89388800	-0.54328600	1.03576700
C	5.84291200	0.61351800	-0.88958000
C	4.93627400	-0.35438900	-0.14989200
H	6.80223500	0.73798700	-0.38199300
H	6.04685900	0.26360300	-1.90550200
H	5.37912400	1.60089300	-0.97108000
C	3.59187300	-0.54753000	-0.83039800
H	5.43503500	-1.32549800	-0.05057900
H	4.77081500	0.00471300	0.87270100
C	2.66145500	-1.48452700	-0.08084600
H	3.09812800	0.42580800	-0.94206800
H	3.75017500	-0.92308200	-1.84926900
H	3.12948200	-2.47244800	0.01462700
H	2.52828800	-1.11075500	0.94188000
C	1.29587800	-1.61548600	-0.73087200
C	0.31900600	-2.44480100	0.08481000
H	1.40131400	-2.03987000	-1.73699900
H	0.87326600	-0.61269400	-0.86072400
C	-1.09311200	-2.38914400	-0.47243000
H	0.66875100	-3.48169800	0.15979300
H	0.30213800	-2.04860300	1.10691900
C	-2.13228800	-2.96241400	0.46642900
H	-1.34366300	-1.33912000	-0.64464000
H	-1.15078300	-2.88002000	-1.44888400
H	-2.22966600	-4.04681000	0.33857400
H	-1.83798600	-2.76504500	1.50410100
O	-3.38158700	-2.32291100	0.19540600
H	-4.06758000	-2.76008100	0.70609200

Table S10. Thy (HBA)/Oct. E = -854.067957594663 a.u.

O	2.61172400	-0.06794500	-1.85003400

C	3.58634900	0.54316500	0.69111900
C	2.20904100	0.96447000	0.23873100
C	3.72977000	0.46194000	2.20257800
C	4.64637000	1.47138400	0.09757100
C	1.77330300	0.66566200	-1.05457000
C	1.32600100	1.67461400	1.04099900
C	-0.33949400	1.78520400	-0.69922900
C	0.52920400	1.06661200	-1.51346100
C	0.07675000	2.07894300	0.59165400
C	-1.67239100	2.23387800	-1.21338200
H	3.76867200	-0.45901900	0.28934100
H	3.66832200	1.44929900	2.66847400
H	4.70462600	0.04287300	2.46006300
H	2.96109000	-0.17349900	2.64857100
H	4.58348100	1.49755600	-0.99117800
H	5.64912900	1.13529800	0.37358600
H	4.51335900	2.48995100	0.47253000
H	1.61748000	1.91438100	2.05635500
H	0.22386100	0.80180100	-2.52341100
H	-0.58261800	2.62555300	1.25687400
H	-1.57698000	3.15546700	-1.79560600
H	-2.12341000	1.48210300	-1.86442800
H	-2.36520800	2.43184600	-0.39419900
H	2.21877300	-0.14856500	-2.72404700
C	-6.60164800	0.64291900	0.12954400
C	-5.30057200	0.32251200	0.84397100
H	-7.27365600	1.23140800	0.75833800
H	-6.41741500	1.21347100	-0.78527700
H	-7.12902600	-0.27174000	-0.15556800
C	-4.34030600	-0.49407500	-0.00427100
H	-4.80749500	1.25386000	1.14706200
H	-5.51275300	-0.22058900	1.77210800
C	-3.03283800	-0.81389500	0.69842100
H	-4.82935700	-1.42741400	-0.31021500
H	-4.12863400	0.05119400	-0.93290000
H	-2.56204000	0.11887100	1.03296700
H	-3.23983800	-1.38936600	1.60926100
C	-2.04659200	-1.57507300	-0.16951900
C	-0.72744400	-1.85476700	0.52860000
H	-1.84746900	-0.99306700	-1.07799900
H	-2.49825000	-2.51649600	-0.50566000
C	0.32118500	-2.45930500	-0.38754300
H	-0.33625600	-0.91338700	0.93231700
H	-0.89943400	-2.51158200	1.39048300

C	1.65799500	-2.65485400	0.30534200
H	-0.02287500	-3.41087800	-0.80663100
H	0.46969600	-1.78272900	-1.23649800
H	1.86531200	-1.77804800	0.93491300
H	1.61990200	-3.52228900	0.97005900
O	2.72395300	-2.88803600	-0.58972700
H	2.83548700	-2.08442200	-1.11092600

Table S11. Cam/Dod. E = -933.737329712808 a.u.

O	-4.00766700	-1.86344000	0.08591400
C	-3.37513100	1.53820900	0.10117700
C	-4.23330600	0.44942900	0.81193900
C	-4.14555300	1.42529400	-1.23810800
C	-5.67560800	0.95769100	0.55385400
C	-5.59760500	1.66970500	-0.81454600
C	-4.02877900	-0.06498800	-1.55935500
C	-4.08476900	-0.68731700	-0.17713200
C	-3.46869400	2.90964500	0.75121800
C	-1.90142900	1.16229300	-0.00439100
C	-3.92467500	0.08362400	2.23709500
H	-3.78666300	2.08396600	-2.03045200
H	-5.96081900	1.64124500	1.35618100
H	-6.39559100	0.13665600	0.56971800
H	-6.30053200	1.25779800	-1.54097600
H	-5.81826200	2.73420200	-0.72480000
H	-3.08528700	-0.34747800	-2.03203900
H	-4.83544400	-0.45084700	-2.18750700
H	-4.49233000	3.24404300	0.91780800
H	-2.97438600	3.65463800	0.12144200
H	-2.95821400	2.90987500	1.71742200
H	-1.37312500	1.88060200	-0.63712000
H	-1.73213800	0.16837300	-0.42210000
H	-1.43064300	1.18947700	0.98148500
H	-4.08093700	0.93545100	2.90297200
H	-4.56834100	-0.73331100	2.56957200
H	-2.89207500	-0.25538500	2.34466300
C	7.03744200	0.72034100	-0.01761200
C	5.62797100	0.26317500	0.31687900
H	7.70633600	0.47893500	0.81818300
H	7.41059500	0.14695100	-0.87565400
C	5.53794500	-1.22434400	0.61904900
H	5.25810400	0.82960800	1.18138300
H	4.96461400	0.51698900	-0.51727700
C	4.15338200	-1.69994000	1.03726300

H	5.86444600	-1.79155700	-0.26174800
H	6.25290700	-1.46478500	1.41436400
H	4.21584900	-2.74882400	1.34937100
H	3.83123700	-1.13988400	1.92439300
C	3.09234800	-1.57617600	-0.04456300
C	1.74250300	-2.13104700	0.37772800
H	3.43339900	-2.09965000	-0.94675000
H	2.97002400	-0.52621200	-0.33213100
C	0.67882700	-1.99928500	-0.69762400
H	1.85598400	-3.18621500	0.65560300
H	1.40982100	-1.61407200	1.28683000
C	-0.66176100	-2.54377300	-0.26458200
H	0.55681600	-0.94617600	-0.97538700
H	0.99383200	-2.52441700	-1.60588800
H	-0.56013500	-3.60460500	0.00841300
H	-1.00309200	-2.01265000	0.63628600
O	-1.58595300	-2.38511400	-1.31813600
H	-2.47372000	-2.53061600	-0.96316700
C	7.13643000	2.20487500	-0.32648900
H	6.46890200	2.44317900	-1.16272700
H	6.76144000	2.77615100	0.53071100
C	8.54972200	2.65063200	-0.65840100
H	8.59498700	3.72006900	-0.87662700
H	9.23040400	2.45211100	0.17431100
H	8.93545000	2.11653600	-1.53137400

Table S12. Hpph (HBD)/Dod. E = -967.227844727 a.u.

C	3.44503600	-1.47181500	-2.14798500
C	3.58502500	-0.95438800	-0.87648500
C	2.58812200	-1.13108800	0.08588600
C	1.42576000	-1.84493800	-0.24425300
C	1.31726500	-2.36102100	-1.53889100
C	2.30191300	-2.18628700	-2.48767800
H	4.23477800	-1.32275200	-2.87606800
H	4.47847000	-0.40524400	-0.60012100
H	0.40704600	-2.90085300	-1.77041000
H	2.18570800	-2.60013100	-3.48173200
C	0.24828600	-2.07355800	0.65240200
C	0.30539000	-1.68927400	2.10769600
C	-0.96234200	-2.03648400	2.85716000
H	1.18707100	-2.16270600	2.55207100
H	0.52710700	-0.61969600	2.16401800
H	-0.87838600	-1.74103900	3.90492500
H	-1.82535900	-1.52946300	2.42225900

H	-1.16688700	-3.10723800	2.81415500
O	2.74709200	-0.60853800	1.31620000
H	3.33056600	0.17471100	1.26358200
O	-0.75588600	-2.57687000	0.19344600
C	-4.94902000	0.93691700	-0.42843100
C	-3.49077800	0.56193100	-0.23228600
H	-5.23422000	1.69398100	0.31343900
H	-5.07343900	1.41539900	-1.40836900
C	-2.54128000	1.74442000	-0.32400200
H	-3.36827600	0.07595400	0.74350400
H	-3.20298100	-0.19302600	-0.97357000
C	-1.08730000	1.34621300	-0.14915800
H	-2.66984600	2.24220500	-1.29342000
H	-2.81137400	2.48881100	0.43585500
H	-0.97564800	0.81486600	0.80367200
H	-0.81434300	0.62103300	-0.92512500
C	-0.11031300	2.50961200	-0.18158300
C	1.32810000	2.03975400	-0.05873800
H	-0.34370200	3.20625500	0.63340900
H	-0.23844200	3.07577400	-1.11262100
C	2.34931400	3.16052100	0.02303800
H	1.41450800	1.41080300	0.83073900
H	1.56676800	1.39277600	-0.91134900
C	3.78126200	2.68032300	-0.01087400
H	2.21914600	3.84519400	-0.82346900
H	2.19071300	3.75058900	0.93212900
H	4.46309600	3.53640100	0.01927200
H	3.96787900	2.12606100	-0.93945800
O	4.04577000	1.82951500	1.11150500
H	4.98909400	1.83858700	1.28672900
C	-5.89621100	-0.24688100	-0.32543400
H	-5.60806400	-1.00257100	-1.06499700
H	-5.77063400	-0.72292600	0.65380200
C	-7.35197600	0.13652200	-0.52511200
H	-8.01212000	-0.73033400	-0.44599600
H	-7.67246500	0.86777000	0.22258200
H	-7.50908200	0.58508600	-1.51028100

Table S13. Hpph (HBA)/Dod. E = -967.222513009 a.u.

C	-0.99338300	-2.69764400	0.16622200
C	-2.25392800	-3.18237300	0.45344900
C	-3.37587800	-2.36769900	0.32610000
C	-3.23618300	-1.03515600	-0.09213300
C	-1.94438000	-0.57453300	-0.37033300

C	-0.83340800	-1.38261900	-0.24960500
H	-0.13382700	-3.35005500	0.26934800
H	-2.38249300	-4.20932800	0.78399600
H	-1.83160400	0.45384100	-0.69183100
H	0.15018700	-0.99053100	-0.47500300
C	-4.35264200	-0.06060800	-0.26839900
C	-5.78674100	-0.51205400	-0.20799400
C	-6.76911400	0.58538900	-0.55573300
H	-5.90029800	-1.38297400	-0.86091300
H	-5.96661000	-0.90684000	0.79726900
H	-7.79219900	0.20973900	-0.49482800
H	-6.67064200	1.43243700	0.12399000
H	-6.59961800	0.96165000	-1.56575500
O	-4.60144900	-2.86238400	0.62011700
H	-4.50662000	-3.77883700	0.89564900
O	-4.10421100	1.11207400	-0.47927700
C	6.70052500	-0.27686300	-0.06421600
C	5.18648900	-0.37686800	-0.13260500
H	7.04518000	0.49637300	-0.76233300
H	6.99715600	0.06709700	0.93470900
C	4.47701600	0.92999100	0.17773700
H	4.89094400	-0.72091700	-1.13178500
H	4.84266400	-1.15040600	0.56580200
C	2.96330800	0.82738700	0.10804600
H	4.77148600	1.27442600	1.17691100
H	4.82006400	1.70369300	-0.52026400
H	2.67029600	0.48355800	-0.89231700
H	2.62089100	0.05266000	0.80638300
C	2.24605100	2.13067200	0.41681900
C	0.73497800	2.00647100	0.34402900
H	2.58243200	2.90483900	-0.28381900
H	2.53976400	2.47865300	1.41525500
C	-0.00563100	3.29684500	0.64700600
H	0.44550000	1.66426800	-0.65483900
H	0.40185700	1.23010900	1.04479800
C	-1.50897000	3.14725100	0.55849900
H	0.25193900	3.65003100	1.65236200
H	0.30980200	4.07919200	-0.05274300
H	-1.99521100	4.07725400	0.88619000
H	-1.83758300	2.35015500	1.24201100
O	-1.87076500	2.84067200	-0.76900300
H	-2.72499200	2.38682200	-0.75009700
C	7.40987500	-1.58432000	-0.37499500
H	7.06423000	-2.35558600	0.32321900

H	7.11231700	-1.92618500	-1.37313400
C	8.92269400	-1.47206800	-0.30370100
H	9.24730000	-1.16198000	0.69360300
H	9.40937000	-2.42340400	-0.53059000
H	9.29565200	-0.72984300	-1.01510200

Table S14. Men (HBA)/Dod. E = -936.140880080132 a.u.

C	2.79332500	-0.58779200	0.33906000
C	2.72916100	-1.10209800	-1.09106800
C	2.32193200	-1.69125900	1.28251500
C	0.83174100	-2.67286900	-0.49912300
C	1.33805600	-1.59524100	-1.44888100
C	0.92175800	-2.17087400	0.93556000
C	4.16063300	0.01502600	0.69988700
C	-0.57894000	-3.10506300	-0.85874000
C	4.14150400	0.64582700	2.08593100
C	5.31665700	-0.96840000	0.57757300
H	2.06314100	0.22999400	0.40761800
H	3.43444100	-1.94075000	-1.19075400
H	2.34271900	-1.32833700	2.31237300
H	3.01539100	-2.53929700	1.23754000
H	1.49559500	-3.54418800	-0.58966100
H	1.33715100	-1.97497500	-2.47863200
H	0.64961600	-0.74249100	-1.42683400
H	0.60888300	-2.96125600	1.62512900
H	0.21078200	-1.34422700	1.06605900
H	4.33888800	0.81876700	-0.02119600
H	-0.63351800	-3.48353100	-1.88271900
H	-0.93459700	-3.89235700	-0.18944600
H	-1.27210400	-2.26174400	-0.77806000
H	4.09677300	-0.10936300	2.87524000
H	5.04668100	1.23477400	2.25085300
H	3.28394200	1.31289500	2.21145800
H	5.20238800	-1.81399200	1.26166200
H	6.25861100	-0.47417300	0.82723300
H	5.41504400	-1.36079300	-0.43655600
C	-5.66159100	-0.27411500	0.34906800
C	-4.48492300	0.68295600	0.43193200
H	-5.30449300	-1.26031500	0.02592700
H	-6.08237300	-0.42135200	1.35180000
C	-3.38932400	0.20177100	1.36949000
H	-4.07708600	0.83311500	-0.57380100
H	-4.83898900	1.66702300	0.76511700
C	-2.22567900	1.17002500	1.53122100

H	-3.82898600	0.00289000	2.35360600
H	-3.00648200	-0.76244900	1.01036600
H	-1.53807000	0.77388900	2.28788500
H	-2.59514800	2.12343200	1.92875300
C	-1.44503500	1.42395100	0.25194100
C	-0.17998500	2.23778200	0.46577800
H	-2.07852300	1.92704800	-0.48610300
H	-1.17314300	0.45825200	-0.19478900
C	0.64781100	2.38603300	-0.79853700
H	-0.43732200	3.22412500	0.87016600
H	0.42884400	1.74835200	1.23760900
C	2.00919800	3.01825300	-0.56483500
H	0.81456700	1.39351900	-1.23096900
H	0.10113400	2.95803200	-1.55539800
H	1.90904900	4.09179800	-0.38015200
H	2.45758000	2.58328500	0.34180300
O	2.87269300	2.87571200	-1.66570200
H	3.00188700	1.92645000	-1.80504400
O	3.13782100	-0.05258500	-1.96548000
H	3.01027700	-0.34597200	-2.87150700
C	-6.75904100	0.19388300	-0.59190400
H	-6.33660400	0.33870000	-1.59299600
H	-7.11318700	1.17971600	-0.26898500
C	-7.92989000	-0.77032100	-0.66628400
H	-8.38955900	-0.90722300	0.31659500
H	-8.70458200	-0.41251900	-1.34822900
H	-7.60693600	-1.75462500	-1.01701300

Table S15. OctA/Dod. E = -932.821998290019 a.u.

O	-1.92264500	0.86518200	1.42989500
O	-2.86272400	1.52850400	-0.48699500
C	-7.70091600	-0.47649500	-0.36435000
C	-6.52087100	-0.51835600	0.59108800
C	-8.91645800	-1.23241100	0.14337500
C	-5.30952300	0.23965900	0.07649100
C	-10.09819000	-1.19020400	-0.81103200
C	-4.14323500	0.18996100	1.04144000
C	-11.30745100	-1.94915800	-0.29360100
C	-2.92740200	0.93067800	0.56849300
H	-7.97575600	0.56838100	-0.55415800
H	-7.39602600	-0.88800000	-1.33445500
H	-6.82582800	-0.10646000	1.56138300
H	-6.24592500	-1.56351800	0.78151700
H	-8.64169100	-2.27781600	0.33276900

H	-9.22082500	-0.82133100	1.11423400
H	-4.99734200	-0.16772700	-0.88993500
H	-5.57595000	1.28351800	-0.11474100
H	-10.37122600	-0.14539000	-0.99895100
H	-9.79227400	-1.60063600	-1.78031000
H	-3.83672500	-0.84193700	1.24560000
H	-4.41538800	0.60544900	2.01806000
H	-11.06880300	-3.00358700	-0.12819200
H	-12.14271100	-1.90401000	-0.99608700
H	-11.65190700	-1.53756300	0.65931700
H	-1.16144200	1.37975100	1.04766800
C	9.18191900	-0.91128100	0.35420700
C	7.92319000	-0.77913400	-0.48558800
H	9.50260300	0.08396700	0.68706900
H	8.95165900	-1.47350800	1.26801200
C	6.77650100	-0.10156500	0.24476600
H	8.15413000	-0.21657900	-1.39886100
H	7.60328700	-1.77446600	-0.81854700
C	5.51862700	0.03239100	-0.59618700
H	6.54441800	-0.66476900	1.15726800
H	7.09670200	0.89318300	0.57891900
H	5.75059500	0.59665400	-1.50809900
H	5.19864700	-0.96221400	-0.93114200
C	4.37233100	0.70908500	0.13523800
C	3.11589900	0.84539100	-0.70791600
H	4.69203400	1.70297700	0.47159300
H	4.13817000	0.14394800	1.04574900
C	1.97367200	1.52259400	0.02942300
H	3.35147500	1.41167700	-1.61740000
H	2.79763300	-0.14902000	-1.04464600
C	0.73283500	1.65201700	-0.81980400
H	1.72726900	0.95656500	0.93488700
H	2.27827200	2.52150100	0.35909800
H	0.95805300	2.22568600	-1.72771900
H	0.38503000	0.65858800	-1.13481300
O	-0.27522100	2.31458400	-0.07246800
H	-1.11066800	2.29606100	-0.57403700
C	10.32954400	-1.58934000	-0.37540800
H	10.55763600	-1.02668800	-1.28810200
H	10.00751400	-2.58336500	-0.70681800
C	11.58241100	-1.71508800	0.47351100
H	11.38833100	-2.30063200	1.37660900
H	12.39207000	-2.20488900	-0.07221700
H	11.94238800	-0.73241600	0.79114100

Table S16. Thy (HBD)/Dod. E = -932.537451597713 a.u.

O	3.09970400	-0.05609900	1.63690200
C	3.50757000	-1.33076100	-0.93681700
C	2.19155500	-1.35102900	-0.19430800
C	3.37292400	-1.59644100	-2.42803100
C	4.48911800	-2.31538200	-0.29955700
C	2.07458700	-0.73303700	1.05854700
C	1.06525500	-2.00323600	-0.68253100
C	-0.22398100	-1.46341200	1.27991800
C	0.88738400	-0.79561600	1.77614700
C	-0.12183200	-2.06366500	0.03057200
C	-1.49199000	-1.53785100	2.07437000
H	3.93778200	-0.32579800	-0.84206500
H	3.04882600	-2.62138700	-2.62721400
H	4.33928100	-1.46126700	-2.91865900
H	2.65681800	-0.91630200	-2.89490800
H	4.61766300	-2.11611800	0.76580200
H	5.46825800	-2.25485600	-0.78227700
H	4.11878800	-3.33839000	-0.40696600
H	1.11237600	-2.47818300	-1.65511900
H	0.84826000	-0.29275900	2.73664300
H	-0.97771000	-2.57888400	-0.39218300
H	-1.48569500	-2.40622900	2.74046500
H	-1.62589000	-0.65091000	2.69670900
H	-2.36249600	-1.63143600	1.42225800
H	3.68180300	0.33545800	0.96589200
C	-5.14536300	-0.01158300	-0.64256700
C	-4.12486400	0.90561600	0.00841600
H	-5.31726100	0.31061300	-1.67734600
H	-4.72952000	-1.02518000	-0.71013600
C	-2.79561400	0.94805500	-0.72461000
H	-4.53797900	1.91977100	0.07693000
H	-3.95506100	0.58043600	1.04266100
C	-1.75515200	1.81829500	-0.04212700
H	-2.39768600	-0.07013200	-0.81456700
H	-2.95508800	1.30084200	-1.75119000
H	-2.12180700	2.85023900	0.02657700
H	-1.62543500	1.47214700	0.99068400
C	-0.40516300	1.78879400	-0.73578100
C	0.67702900	2.53670100	0.02377500
H	-0.49883200	2.19086600	-1.75215800
H	-0.09161600	0.74426000	-0.84409100
C	2.05852400	2.31342600	-0.56720700

H	0.44040900	3.60667500	0.07110900
H	0.68258900	2.17434200	1.05849700
C	3.17939500	2.80311000	0.32403300
H	2.19182000	1.23763100	-0.70830300
H	2.13901300	2.76303800	-1.56173600
H	3.39149400	3.86457800	0.15077400
H	2.89353800	2.67826000	1.37520600
O	4.34330600	2.01984400	0.05037400
H	5.08636900	2.39604000	0.52880800
C	-6.47326200	-0.06367600	0.09417100
H	-6.88820300	0.94883900	0.15930000
H	-6.29811000	-0.38432700	1.12774800
C	-7.48282500	-0.98823100	-0.56323500
H	-8.42804000	-1.00865000	-0.01595800
H	-7.69829300	-0.67044500	-1.58731400
H	-7.10420800	-2.01320100	-0.61113900

Table S17. Selected energetic quantities for 1-octanol associates.

	$\Delta G_{1\text{-octanol}}$, kcal/mol	E_e, atomic units
**Hpph (HBD, -OH)**	**23.4**	**-888.759338**
Hpph (HBA, =O)	18.9	-888.754017
Hpph (HBA, -OH)	16.1	-888.749956
**Men (HBA)**	**20.3**	**-857.672334**
Men (HBD)	18.5	-857.671152
**Thy (HBD)**	**25.0**	**-854.068809**
Thy (HBA)	23.9	-854.067958

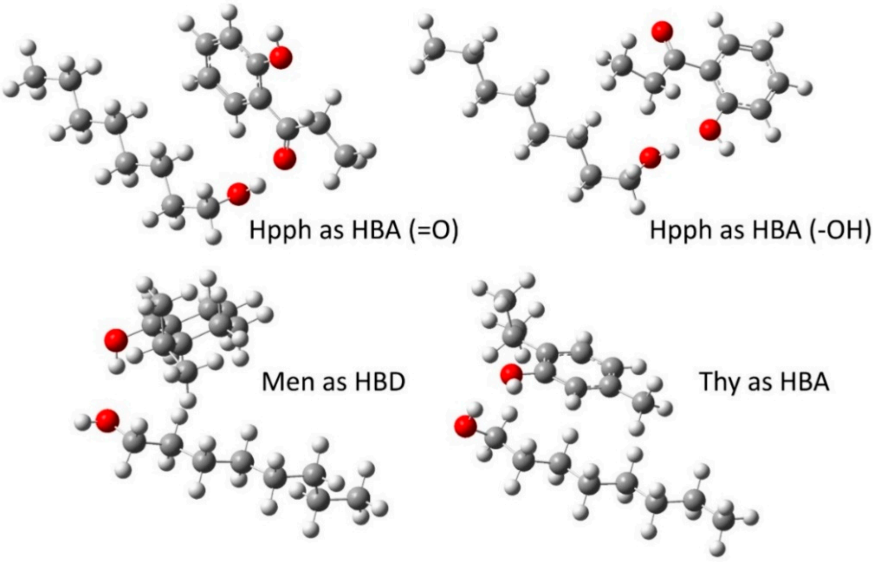

Figure S17. Relaxed structures of octanol's complexes with Hpph as HBA, Men as HBD and Thy as HBA.
